# Fluorescence Imaging and Photodynamic Inactivation of Bacteria Based on Cationic Cyclometalated Iridium(III) Complexes with Aggregation‐Induced Emission Properties

**DOI:** 10.1002/adhm.202100706

**Published:** 2021-07-23

**Authors:** Po‐Yu Ho, Sin‐Ying Lee, Chuen Kam, Junfei Zhu, Guo‐Gang Shan, Yuning Hong, Wai‐Yeung Wong, Sijie Chen

**Affiliations:** ^1^ Ming Wai Lau Centre for Reparative Medicine Karolinska Institutet Hong Kong P. R. China; ^2^ Institute of Functional Materials Chemistry and National & Local United Engineering Lab for Power Battery Faculty of Chemistry Northeast Normal University Changchun 130024 P. R. China; ^3^ Department of Chemistry and Physics La Trobe Institute for Molecular Science La Trobe University Melbourne Victoria 3086 Australia; ^4^ Department of Applied Biology and Chemical Technology The Hong Kong Polytechnic University Hung Hom Hong Kong P. R. China

**Keywords:** aggregation‐induced emission, bacteria, iridium(III) complexes, photodynamic inactivation, photosensitizers

## Abstract

Antibacterial photodynamic therapy (PDT) is one of the emerging methods for curbing multidrug‐resistant bacterial infections. Effective fluorescent photosensitizers with dual functions of bacteria imaging and PDT applications are highly desirable. In this study, three cationic and heteroleptic cyclometalated Ir(III) complexes with the formula of [Ir(CˆN)_2_(NˆN)][PF_6_] are prepared and characterized. These Ir(III) complexes named **Ir(ppy)_2_bP**, **Ir(1‐pq)_2_bP,** and **Ir(2‐pq)_2_bP** are comprised of three CˆN ligands (i.e., 2‐phenylpyridine (ppy), 1‐phenylisoquinoline (1‐pq), and 2‐phenylquinoline (2‐pq)) and one NˆN bidentate co‐ligand (bP). The photophysical characterizations demonstrate that these Ir(III) complexes are red‐emitting, aggregation‐induced emission active luminogens. The substitution of phenylpyridine with phenylquinoline isomers in the molecules greatly enhances their UV and visible‐light absorbance as well as the photoinduced reactive oxygen species (ROS) generation ability. All three Ir(III) complexes can stain both Gram‐positive and Gram‐negative bacteria efficiently. Interestingly, even though **Ir(1‐pq)_2_bP** and **Ir(2‐pq)_2_bP** are constitutional isomers with very similar structures and similar ROS generation ability in buffer, the former eradicates bacteria much more effectively than the other through white light‐irradiated photodynamic inactivation. This work will provide valuable information on the rational design of Ir(III) complexes for fluorescence imaging and efficient photodynamic inactivation of bacteria.

## Graphical abstract

Cationic and cyclometalated iridium complexes are designed, synthesized, and characterized. These phosphors exhibit aggregation‐induced emission (AIE) property and reactive oxygen species generation capability upon light excitation. Bacteria can be stained by treating these AIE‐active phosphors under fluorescence microscope, and the structural changes of the congeners endow different degrees of photodynamic inactivation against bacteria.

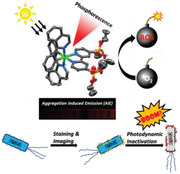

## 1 Introduction

Pathogenic infection is one of the leading causes of death worldwide and bacterial pathogens account for many lethal infectious diseases if appropriate medicines are not prescribed. It was not until the discovery of antibiotics in the 19th century that acute bacterial infections could be cured and the death rate of bacterial infections has dramatically dropped. However, the emerging multidrug‐resistant (MDR) bacteria, such as *Clostridioides difficile*,^[^ ^1^ ^]^ carbapenem‐resistant *Enterobacteriaceae*,^[^ ^2^ ^]^ and carbapenem‐resistant *Acinetobacter*,^[^ ^3^ ^]^ have posed a real threat to human being in both developed and developing countries since the 1960s.^[^ ^4^ ^]^ Discovery and development of next‐generation antibiotics to combat MDR bacteria are ongoing but still need extensive research and pre‐clinical studies.^[^ ^5^ ^]^

To selectively kill bacteria, one of the interesting approaches researchers are now working on is the photodynamic inactivation of bacteria. Photosensitizers are exploited as “drugs” through type I or type II reactive oxygen species (ROS) generation mechanisms upon photoexcitation.^[^ ^6^ ^]^ In order to achieve the specificity of this medical treatment, the photosensitizers are activated upon harmless and pre‐calculated light illumination (light dose) which is fully controllable in a spatiotemporal manner, thus minimizing the unnecessary side‐effects originated from the photosensitizers in the surrounding tissues.^[^ ^7^ ^]^ The more promising strategy is to develop a photosensitizer which fully discriminates the pathogenic bacteria from healthy cells and tissues, hence further maximizing the safety of the medical treatments.^[^ ^8^ ^]^ Overall, this treatment framework resembles the image‐guided photodynamic therapy (PDT) against cancers and solid tumors, which is an established clinical practice and several examples of PDT studies have been successfully approved by the US Food and Drug Administration.^[^ ^9^ ^]^

Considerable number of research works, which reported *heavy element‐free* organic fluorophores with strong photoinduced ROS generation capability, have been conducted over the last decade. These pure organic photosensitizers^[^ ^10^ ^]^ primarily possess a rather narrow singlet–triplet energy gap (∆*E*_ST_ ≤ 0.2 eV) as compared to other ordinary highly *π*‐conjugated small molecules. This feature enables the energetically favorable singlet‐to‐triplet excited state transition as well as energetically unfavorable reverse intersystem crossing upon photoexcitation of the molecules at room temperature even though the molecules are heavy‐element‐free.^[^ ^11^ ^]^ To design and prepare molecules with the abovementioned attribute, researchers find that if the highest occupied molecular orbital (HOMO) and lowest unoccupied molecular orbital (LUMO) of an organic fluorophore is less overlapping with each other, the more likely the fluorophore will have a lower ∆*E*_ST_ and a higher triplet photosensitizing potency.^[^ ^12^ ^]^ This also implies that these appealing fluorophores will have a highly twisting, rotatable, and non‐coplanar molecular skeleton. Coincidently, these structural features are common grounds for aggregation‐induced emission (AIE) luminogens.^[^ ^13^ ^]^ Also, the non‐radiative transition pathways of AIE luminogens in the aggregated state are theoretically inhibited, so the conversion efficiency from the ground state to triplet excited state upon photoexcitation is up‐regulated in whole, hence further promoting the ROS generation. Therefore, some of the reported AIE emitters are also efficient triplet photosensitizers and have been widely applied in many investigations regarding the photodynamic inactivation of cancers cells and bacteria in vitro and in vivo, especially for image‐guided PDT studies.^[^ ^14^ ^]^

On the other hand, phosphorescent transition metal complexes have been thoroughly investigated as the photosensitizers for PDT against tumors and cancers, due to the inherent and highly‐guaranteed photosensitization capability.^[^ ^15^ ^]^ In principle, there are fewer molecular design restrictions in red‐shifting and intensifying the absorption peaks of the photosensitizers (e.g., simply building longer *π*‐conjugation with higher degree of orbital overlapping in the ligands). Until recently, several basic research investigations demonstrate that photoluminescent transition metal complexes can inhibit the growth of bacteria (including drug‐resistant bacteria) as antibiotics or light‐activated antibiotics in addition to common organic antibiotics.^[^ ^16^ ^]^ In 2019, Collins and co‐workers reported the preparation of multi‐nuclear Ru(II) bipyridine complex species and their antimicrobial activities against six strains of bacteria based on minimum bactericidal concentrations.^[^ ^17^ ^]^ In the same year, Thomas and co‐workers investigated the antimicrobial activity of four dinuclear Ru(II) complexes with the same rigid and planar bridging ligand (tetrapyrido[3,2‐*a*:2′,3′‐*c*:3″,2″‐*h*:2‴,3‴‐*j*]phenazine (tpphz)) against *Escherichia coli* *(* *E. coli*) and the *Enterococcus faecalis* strain V583, and super‐resolution stimulated emission depletion nanoscopy was used to image the localization of the phosphorescent emitters.^[^ ^18^ ^]^ In 2020, Frei et al. reported three Re(I) tricarbonyl complexes constituted of three different NˆNˆN tridentate ligands, and one of the chemical species could be light‐activated and subsequently exhibited a dual‐mode antibacterial activity against both Gram‐positive and Gram‐negative bacteria, even including several drug‐resistant bacterial strains.^[^ ^19^ ^]^

Given the above successful examples of transition metal complexes in bacterial photodynamic inactivation, these studies stimulate our curiosity whether phosphorescent Ir(III) complexes can be exploited to act as effective antibacterial agents despite only a few of them have been reported.^[^ ^20^ ^]^ Among the diversified molecular structures of Ir(III) complexes, we consider the [Ir(CˆN)_2_(NˆN)]^+^ framework is of particular interest due to its highly tunable photophysical properties, cationic nature, and ease of preparation.^[^ ^21^ ^]^ Because cationic species are relatively more potent to interact with the negatively charged phospholipids and proteins of bacterial envelope, as compared to non‐charged species in a general consideration.^[^ ^22^ ^]^ And this general molecular structure is well studied because of its rich photophysics, photochemistry, and photosensitizing ability in ambient environment; therefore Ir(III) complexes are involved in a lot of optical applications, including photocatalysis, bioimaging, analytical assays, PDT against cancer, electrochemiluminescence, and light‐emitting devices.^[^ ^23^ ^]^

**Figure 1:**
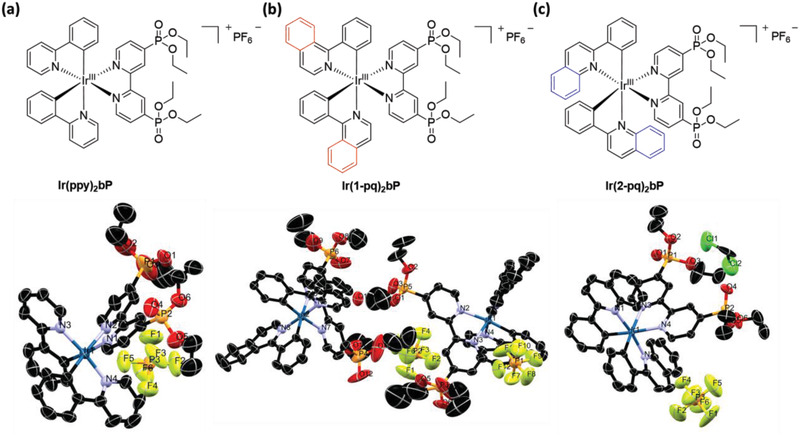
Chemical structures (top) and corresponding X‐ray crystal structure diagrams (bottom) of a) **Ir(ppy)_2_bP**, b) **Ir(1‐pq)_2_bP**, and c) **Ir(2‐pq)_2_bP**, respectively. The oxygen atom of the H_2_O molecule is denoted as “O1W” for the crystal structure diagram of **Ir(1‐pq)_2_bP**. The hydrogen atoms are omitted for clarity. Ellipsoids are plotted at the 40% confidence level.

Herein, three cationic and heteroleptic cyclometalated Ir(III) complexes (i.e., **Ir(ppy)_2_bP**, **Ir(1‐pq)_2_bP**, and **Ir(2‐pq)_2_bP**, see structures in **Figure** **1**) comprised of three CˆN ligands (i.e., 2‐phenylpyridine (ppy), 1‐phenylisoquinoline (1‐pq), and 2‐phenylquinoline (2‐pq)) and one NˆN bidentate co‐ligand (i.e., tetraethyl [2,2″‐bipyridine]‐4,4″‐diylbis(phosphonate) (bP))^[^ ^24^ ^]^ were designed, synthesized, and well characterized. The specific diimine co‐ligand was chosen to lower the energy levels of LUMO among the metal complexes, hence reducing the HOMO–LUMO gap (i.e., equivalent to increasing UV–Vis absorption wavelength) of the metal complexes due to the substituted electron‐withdrawing phosphonate esters.^[^ ^25^ ^]^ In addition to the fundamental understanding of the photophysical properties and photochemistry, these three red photosensitizers were minutely examined in fluorescence imaging and visible light photodynamic inactivation of bacteria.

## 2 Results and Discussion

### 2.1 Synthesis and Photophysical Properties of Ir(III) Complexes

The straightforward two‐step synthetic routes for the three cationic Ir(III) complexes are shown in Scheme S1, Supporting Information. The commercially available cyclometalated ligands were first reacted with the Ir(III) trichloride hydrate salt to prepare the three poorly soluble dimers respectively under the classical protocol first described by Nonoyama et al.^[^ ^26^ ^]^ The insoluble dimers were then chemically converted into the target metal complexes by reacting with the electron‐withdrawing NˆN bidentate co‐ligand bP and an excess amount of hexafluorophosphate salt, and the reaction mixtures were purified using silica gel‐based column chromatography. All three Ir(III) complexes were air‐stable and structurally characterized by matrix‐assisted laser desorption ionization time‐of‐flight (MALDI‐TOF) mass spectrometry as well as ^1^H and ^13^C NMR spectroscopy.

In addition, the products could be readily recrystallized using diethyl ether/dichloromethane solvent system under slow diffusion, while single crystals suitable for X‐ray diffraction analyses were performed to render the exact 3D molecular structures (see Figure 1; selected bond lengths (Å) and angles (°) of the three species were collected in Table S4–S6, Supporting Information). Compared to other previously reported and structurally similar cationic Ir(III) complexes (e.g., bipyridine‐based derivatives) in the literature, the chelating bonding and slightly‐distorted octahedral geometry of these new congeners did not show a significant difference.^[^ ^27^ ^]^

**Figure 2:**
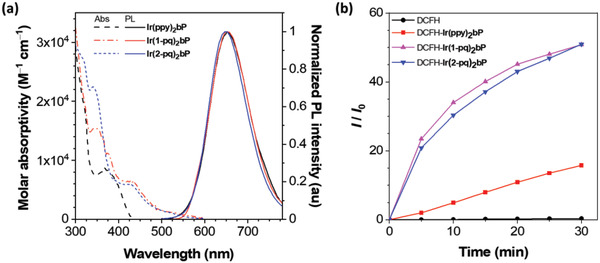
a) UV–Vis absorption spectra (dotted line) and normalized PL spectra (solid line) for the Ir(III) complexes in deaerated acetonitrile. b) Fold of increase (*I*/*I*_0_) of PL intensity (excitation at 488 nm and emission at 530 nm) of the solution mixtures of 1 µM DCFH with and without 10 µM Ir(III) complex upon white light illumination over time.

The UV–Vis absorption spectra and photoluminescence (PL) spectra of the metal complexes in solutions are depicted in **Figure** **2**a and the photophysical properties are summarized in **Table** **1**. Both constitutional isomers **Ir(1‐pq)_2_bP** and **Ir(2‐pq)_2_bP** exhibited a red‐shifted absorption maximum at ≈430 nm and an absorption tail up to 600 nm while **Ir(ppy)_2_bP** only weakly absorbed in the blue light region. However, all three metal complexes were photoluminescent at ≈650 nm in the deaerated solutions upon photoexcitation and the excitation was rooted in the *dπ* → π* singlet metal/ligand‐to‐ligand charge transfer (^1^MLLCT) excited state (i.e., the lowest energy absorption band). This intuitive assignment was supported by the density functional theory^[^ ^28^ ^]^ calculations (see Figure S17, Supporting Information and calculation details in Supporting Information). The data showed that the HOMO was localized over the Ir(III) *d* orbitals and *π* orbitals on the CˆN ligand, and the LUMO was localized over the π* orbitals on the diimine‐based moiety in each Ir(III) complex. Theoretically, swift intersystem crossing from ^1^MLLCT to triplet MLLCT (^3^MLLCT) excited state occurs due to the strong spin‐orbit coupling given by the heavy Ir(III) metal center.^[^ ^29^ ^]^ The triplet state (T_1_)‐to‐ground state (S_0_) radiative decay (i.e., phosphorescence centered at ≈650 nm) is either originated from an entirely triplet ligand‐centered lowest‐lying excited state (^3^LC) or a mixed ^3^MLLCT/^3^LC excited state after an internal conversion, in which the T_1_ composition nature is dependent of the relative energies of both ^3^MLLCT and ^3^LC excited states.^[^ ^25^ ^]^

**Table 1:** Photophysical properties of **Ir(ppy)_2_bP**, **Ir(1‐pq)_2_bP**, and **Ir(2‐pq)_2_bP** in different solvent systems under different conditions and solid‐state

	CH_3_CN^a)^		CH_3_OH		CH_3_OH:Et_2_O^a)^, ^c)^	Thin film
Complex	*λ*_abs_ (*ε* [M^−1^ cm^−1^]) [nm]	*λ*_em_ [nm]	*Φ*_Δ_ ^b)^ [%]	*λ*_em_ ^a)^ (*Φ*_PLQY_ ^a)^ [%]) [nm]	*λ*_em_ (*Φ*_PLQY_ [%]) [nm]	*τ* (*λ*_em_ [nm]) [µs]
**Ir(ppy)_2_bP**	373 (8 290)	652	50.4	667 (0.2)	637 (12.2)	9.77 (559)
**Ir(1‐pq)_2_bP**	344 (15 400), 374 (11 200), 429 (6 460)	652	29.6	655 (0.8)	602 (5.6)	9.60 (564)
**Ir(2‐pq)_2_bP**	341 (22 400), 426 (5 900)	650	30.3	668 (0.3)	642 (11.0)	9.66 (564)

^a)^ Measured in deaerated solution

^b)^ Measurement in ambient solution

^c)^ *f*_DE_ of 90% for **Ir(ppy)_2_bP**, *f*_DE_ of 99% for **Ir(1‐pq)_2_bP**, and *f*_DE_ of 99% for **Ir(2‐pq)_2_bP**.

Conventionally, structurally similar Ir(III) complexes are classified as phosphors which exhibit phosphorescence with relatively short triplet excited state lifetimes (≥100 ns). Therefore they enable the photosensitization of triplet excited state of dioxygen, hence generating singlet oxygen (^1^O_2_) efficiently.^[^ ^30^ ^]^ To qualitatively examine the photosensitization function of the Ir(III) complexes, a fluorescence assay based on 2″,7″‐dichlorodihydrofluorescin (DCFH, as a ROS reporter; see molecular structure in Figure S1, Supporting Information) was adopted.^[^ ^31^ ^]^ As shown in Figure 2b, an increase in the PL intensity ratio of DCFH is accompanied by ROS production over time in the presence of Ir(III) complexes upon light illumination. As a result, all three Ir(III) complexes were active in ROS generation by photoexcitation. Furthermore, the activity of ROS production remained in the phosphors‐stained bacteria (see the results in Figure S15, Supporting Information), and the relative singlet oxygen quantum yield (*Φ*_Δ_) of the metallophosphors in methanol were computed using tris(bipyridyl)ruthenium(II) chloride (see the molecular structure in Figure S1, Supporting Information) as a reference standard and summarized in Table 1 (see calculation details in Figure S11–S14, Supporting Information). The *Φ*_Δ_ of **Ir(ppy)_2_bP, Ir(1‐pq)_2_bP**, and **Ir(2‐pq)_2_bP** in ambient methanol were 50.4%, 29.6%, and 30.3%, respectively. All the data indicate that the Ir(III) complexes are efficient photosensitizers which can be further applied for photodynamic inactivation against bacteria.

**Figure 3:**
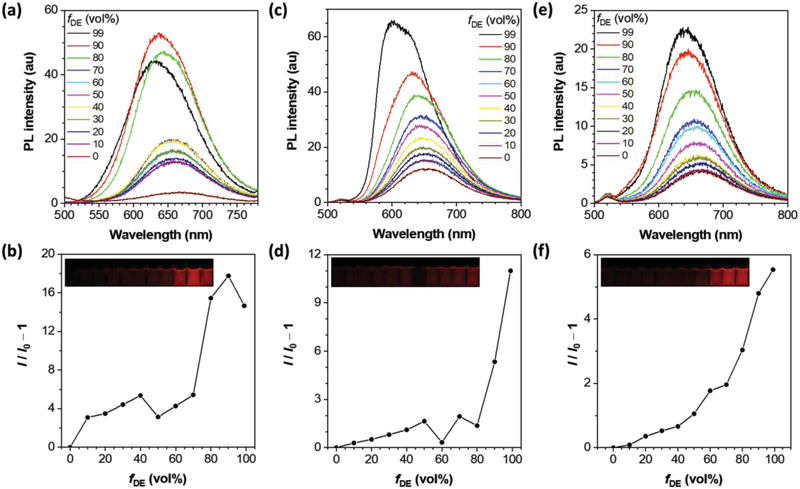
PL spectra of the corresponding solutions (10 µM) and corresponding plots of the relative emission intensity for a–b) **Ir(ppy)_2_bP**, c–d) **Ir(1‐pq)_2_bP**, and e–f) **Ir(2‐pq)_2_bP**, respectively. The emission wavelengths taken are 637, 602, and 642 nm for b,d,f), respectively. Insets: images of methanol and diethyl ether mixture solutions (from left to right: increasing fraction of diethyl ether (*f*_DE_ in volume%, 0 to 99%)) under UV 365 nm excitation.

During the course of the fundamental photophysical characterizations, the so‐called “fluorogenicity” of the Ir(III) complexes in different solutions and solid‐state were visible to the naked eyes with the aid of a UV‐light pen. Therefore, the AIE attributes of the metal complexes were also investigated. Among the binary solvent systems studied, the three Ir(III) complexes exhibited significant fluorescence intensity change in the mixtures of methanol and diethyl ether with different volume fractions (see **Figure** **3** and Table 1), and the cationic species were highly soluble in hydrophilic methanol but completely insoluble in hydrophobic diethyl ether. When the fraction of the poor solvent was increased from 0% to 70%, all three complexes exhibited rather minor enhancement in the PL intensity. However, when the fraction of the poor solvent was further increased to 80% or above, there was a relatively prominent increase in the PL intensity as well as a moderate hypsochromic shift in wavelength because of the nano‐aggregate formation and conversion to the AIE state.^[^ ^32^ ^]^ When the fraction of poor solvent was further approaching unity, the precipitation of the emitters could be observed in the standing still solutions with the naked eye, therefore a clear downturn of the relative emission intensity was recorded in the case of **Ir(ppy)_2_bP** (Figure 2b). Quantitatively, the PL quantum yield (*Φ*_PLQY_) of these three species under deaerated condition were reported in Table 1. In methanol, the *Φ*_PLQY_ of the metal complexes were all below 1%, while the maximal *Φ*_PLQY_ of **Ir(ppy)_2_bP**, **Ir(1‐pq)_2_bP**, and **Ir(2‐pq)_2_bP** in the AIE state increased to 12.2%, 5.6%, and 11.0%, respectively. In addition, the emission lifetimes of the complexes in solid‐state (thin‐films being spin‐coated on quartz plates) obtained from time‐resolved single‐photon counting were 9.77, 9.60, and 9.66 µs for **Ir(ppy)_2_bP**, **Ir(1‐pq)_2_bP**, and **Ir(2‐pq)_2_bP**, respectively (see Table 1 and Figure S18–S20, Supporting Information).

### 2.2 Bacterial Imaging and Photodynamic Inactivation

**Figure 4:**
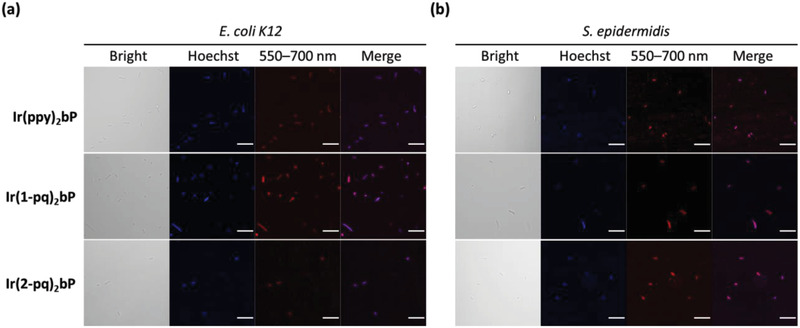
Bright‐field and fluorescence images of a) *E. coli* K‐12 and b) *S. epidermidis* co‐stained with 1 µg mL^−1^ of Hoechst 33 342 and 10 µM of **Ir(ppy)_2_bP**, **Ir(1‐pq)_2_bP**, or **Ir(1‐pq)_2_bP**, respectively. Scale bar: 10 µm.

Because of the electrostatic interaction between the anionic surface of bacteria and the cationic Ir(III) complexes, we envisaged these Ir(III) complexes would form aggregates on the bacterial surface and their AIE properties can be utilized for bacterial imaging under fluorescence microscopy.^[^ ^22^ ^]^ Generally, bacteria can be classified into Gram‐positive bacteria and Gram‐negative bacteria based on their reactions to the Gram staining,^[^ ^33^ ^]^ in which the Gram‐negative bacteria is surrounded by an additional lipopolysaccharide layer as the outermost component and the Gram‐positive bacteria is instead protected by a peptidoglycan layer as the outermost component.^[^ ^33a^ ^]^ In this study, the *E. coli* K‐12 strain and *Staphylococcus epidermidis* (*S. epidermidis*) were selected as the illustrative examples of Gram‐negative bacteria and Gram‐positive bacteria, respectively, in bacterial fluorescence imaging (see **Figure** **4**).

In order to evaluate the staining capability of these three cationic Ir(III) complexes in live bacteria, Gram‐negative and Gram‐positive bacteria were respectively co‐stained by Ir(III) complexes and Hoechst 33 342. Hoechst 33 342 is a blue‐emissive and cell‐permeable fluorophore that can selectively light‐up by the presence of DNA in bacteria. Compared with the signal from Hoechst 33 342, we could observe that all three cationic Ir(III) complexes fluorescently and explicitly stained both *E. coli* K‐12 and *S. epidermidis* indistinguishably in bacterial imaging using confocal laser scanning microscopy, even though these two types of bacteria possess different cell wall structures.

**Figure 5:**
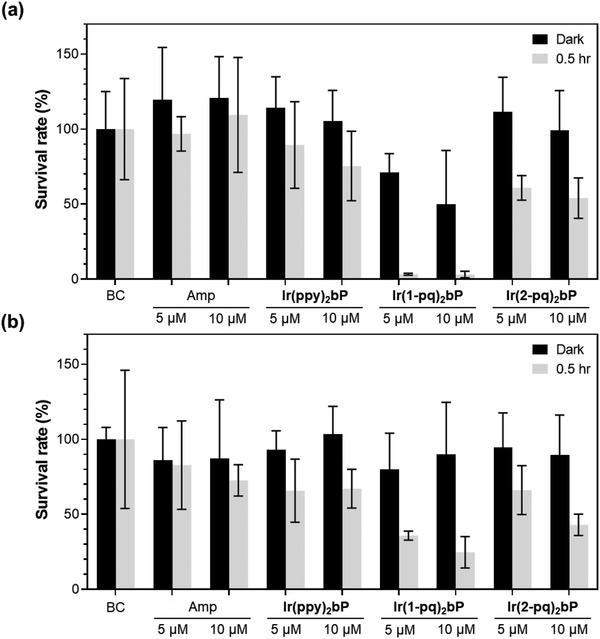
Plate‐count results of a) *E. coli* K‐12 and b) *S. epidermidis* treated with the BC, ampicillin (Amp), or Ir(III) complexes (5 or 10 µM) in darkness or upon white light illumination (LED with a power of 134 mW cm^−2^) for 0.5 h.

**Figure 6:**
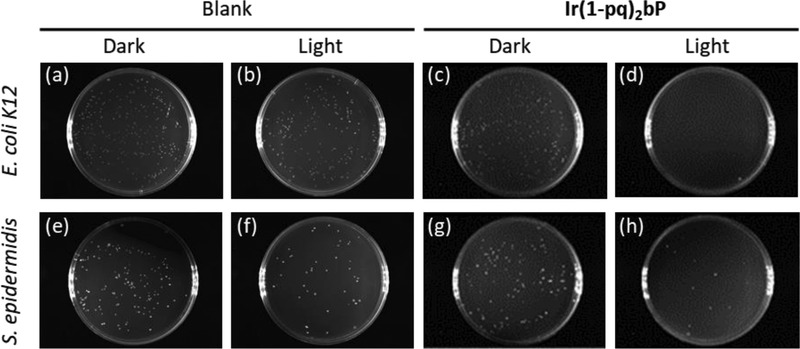
Representative plate images of a–d) *E. coli* K‐12 and e–h) *S. epidermidis* incubated overnight without or with white light illumination for 1 h in the presence of a,b,e,f) the BC or c,d,g,h) **Ir(1‐pq)_2_bP** (10 µM).

To evaluate the efficacy of three Ir(III) complexes against both *E. coli* K‐12 and *S. epidermidis* in photodynamic inactivation, plate‐count method was adopted. In addition to treating the bacteria with these three cationic Ir(III) complexes in dark condition or under white light‐emitting diode (LED) for illumination, blank control (BC) and ampicillin^[^ ^34^ ^]^ (Amp, an antibiotic that is effective against many types of Gram‐positive and Gram‐negative bacteria)‐treated bacteria were also included for comparison. Furthermore, 5 or 10 µM of the potential photosensitizers were used to have a *preliminary* understanding of their cytotoxicity in the dark condition and phototoxicity to the bacteria. The corresponding bacterial survival rates (%) are depicted in **Figure** **5** (see the full set of colony formation raw data in Figure S16, Supporting Information), and the representative images of **Ir(1‐pq)_2_bP**‐treated or BC of *E. coli* K‐12 and *S. epidermidis* grown overnight on agar plates are shown in **Figure** **6**.

In the dark condition, all three metal complexes did not exhibit significant antibacterial activity against *S. epidermidis*. While both **Ir(ppy)_2_bP** and **Ir(2‐pq)_2_bP** were not cytotoxic to *E. coli* K‐12, only **Ir(1‐pq)_2_bP** demonstrated minor and discernible antibacterial activity against this Gram‐negative bacterium at both 5 and 10 µM. Under white light illumination, it was clear that white light could inhibit the colony growth of both *S. epidermidis* and *E. coli* K‐12. However, the complete eradication of both bacteria was not observed. This result is consistent with the earlier investigations that *endogenous* photosensitizers are naturally present in the bacteria.^[^ ^35^ ^]^ Therefore, there will be a necessity to develop *exogenous* photosensitizers with desired photodynamic inactivation capability, which can be widely exploited to treat antibiotic‐resistant bacterial infections effectively.

Comparing these three Ir(III) species, it was also clear that **Ir(ppy)_2_bP** at the concentration of 5 or 10 µM could not function as an efficient photosensitizer against both types of bacteria (Figure 5), which can be explained by its weak UV–Vis absorption in the mid‐visible light region. In addition, one of the red‐shifted structural isomers, **Ir(2‐pq)_2_bP**, at both concentrations did show notable inhibitory effect towards *E. coli* K‐12 but *not S. epidermidis* on top of the effect of white light illumination. However, another structural isomer **Ir(1‐pq)_2_bP** at the concentration of 10 µM with similar UV–Vis absorption peak wavelength and molar absorptivity as well as *Φ*_Δ_ could almost eliminate the colony formation of *E. coli* K‐12 and prominently inhibited the growth of *S. epidermidis* after illumination for one and a half hour. Therefore, **Ir(1‐pq)_2_bP** demonstrated better performance than **Ir(2‐pq)_2_bP** as a photosensitizer to kill both types of bacteria photodynamically. This implies **Ir(1‐pq)_2_bP** may better interact with or more likely to aggregate on (or even internalized into) both types of bacteria.

The discovery of structural isomer effect towards the cationic Ir(III) complexes in acting as photosensitizers to deactivate bacteria photodynamically can become a molecular design strategy for other phosphorescent metal complexes as well as organic photosensitizers. In this sense, structural engineering of the 3D ligands of iridium or other platinum‐group metal complexes will further fine‐tune the physical interaction between photosensitizers and bacteria. This may be a feasible approach to ameliorate the photosensitization performance along with varied photochemistry aspects.

**Figure 7:**
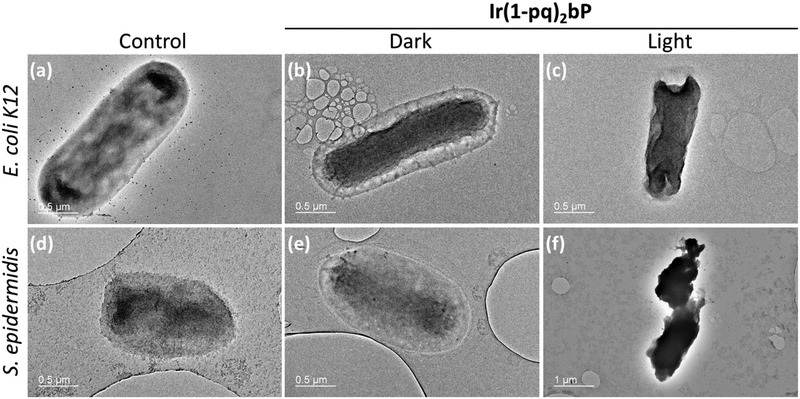
TEM images of a–c) *E. coli* K‐12 and d–f) *S. epidermidis* incubated with b,c,e,f) **Ir(1‐pq)_2_bP**, followed by b,e) keeping in darkness or c,f) white light illumination (134 mW cm^−2^) for 30 min. a,d) The control is bacteria without incubation with Ir(III) complex. Scale bar: 0.5 µm for panel (a–e) or 1 µm for panel (f).

We also investigated the mechanism of bacterial photodynamic inactivation driven by **Ir(1‐pq)_2_bP** through ultrastructural examination of the bacterial morphology. In this aspect, transmission electron microscopy (TEM) was used to probe the significant morphological changes to both types of bacteria in the presence of **Ir(1‐pq)_2_bP** (**Figure** **7**). Without light illumination and **Ir(1‐pq)_2_bP** treatment (Figure 7a,d), *E. coli* K‐12 and *S. epidermidis* exhibited rod‐shape and oval‐/spherical‐shape, respectively, with a smooth and intact bacterial envelope. The bacterial morphology was retained when both types of bacteria were treated with **Ir(1‐pq)_2_bP** in the dark condition (Figure 7b, e). However, the bacteria lost their original shapes and the bacterial envelopes became wrinkled and ruptured, when the bacteria were treated with **Ir(1‐pq)_2_bP** under white light illumination (Figure 7c,f). Therefore, it was speculated that **Ir(1‐pq)_2_bP** can *partially* disintegrate the double or multi‐layered cell wall structure of both Gram‐positive and Gram‐negative bacteria through photodynamic inactivation process, leading to the irreversible release of intracellular materials (e.g., ribosomes, plasmids, and food granules) to the external environment.

## 3 Conclusion

In this study, three cationic and heteroleptic cyclometalated Ir(III) complexes, comprised of different CˆN ligands and the same NˆN bidentate co‐ligand, were prepared and characterized. As compared to **Ir(ppy)_2_bP**, the other two entities with two more phenyl rings in their CˆN ligands (i.e., **Ir(1‐pq)_2_bP** and **Ir(2‐pq)_2_bP**) showed red‐shifted absorption bands in the visible spectrum, which were beneficial for visible‐light PDT. These three Ir(III) complexes were red‐emissive and displayed AIE characteristics. Though they were similar in chemical structures, they had different photoinduced ROS generation abilities. These three Ir(III) complexes could be utilized to stain both Gram‐positive and Gram‐negative bacteria (i.e., *S. epidermidis* and *E. coli*, respectively) for fluorescence imaging.

Among the three Ir(III) complexes, **Ir(1‐pq)_2_bP** effectively eradicated both bacteria through white light‐based photodynamic inactivation in vitro and the other two structural congeners exhibited weaker bacteria‐killing capability under the same condition. Intriguingly, the pair of constitutional isomers demonstrated disparate performances in extirpating the bacteria whereas **Ir(1‐pq)_2_bP** and **Ir(2‐pq)_2_bP** indeed resembled each other in their UV–Vis absorption spectra and ROS generation efficiencies in solutions. This implied the 3D physical interaction between photosensitizers and bacteria played a critical role in the photodynamic inactivation process. The TEM investigation fully supported the plate‐count results regarding the inhibition of colony formation. This indicated the significant and irreversible disruption of bacterial envelope driven by the Ir(III) complex for both Gram‐positive and Gram‐negative bacteria upon photodynamic inactivation.

Overall, this investigation reveals that phosphorescent Ir(III) complexes are competent in simultaneous bacterial detection, fluorescence imaging, and photodynamic inactivation. Especially, the discovery of photodynamic inactivation improvement driven by the structural isomer effect and thus the physical interaction between photosensitizers and bacteria renders a new dimension in the molecular design strategies for photosensitizers. In this regard, the construction of libraries of various transition metal complexes using structural isomerism as one of the design elements sounds like a promising approach, in order to maximize the photocytotoxicity against bacteria. With a dedicated ligand design and molecular engineering to modulate their functions, cationic Ir(III) complexes are believed to have full potential in clinical phototherapy for different MDR bacterial infections.

## Conflict of Interest

The authors declare no conflict of interest.

## Supporting information

Supporting Information

## Data Availability

The data that supports the findings of this study are available in the supplementary material of this article.
