# Supplementary material for: Fluorescence Imaging and Photodynamic Inactivation of Bacteria Based on Cationic Cyclometalated Iridium(III) Complexes with Aggregation‐Induced Emission Properties
Source: Adv Healthc Mater. 2021 Jul 23;10(24):2100706. doi: 10.1002/adhm.202100706 (PMC11468684; doi:10.1002/adhm.202100706)
Supplement: Supplementary file 1 — Supporting Information [file ADHM-10-2100706-s001.pdf]

© 2021 Wiley-VCH GmbH

ADVANCED HEALTHCARE MATERIALS

Supporting Information

for *Adv. Healthcare Mater.*, DOI: 10.1002/adhm.202100706

Fluorescence Imaging and Photodynamic Inactivation of Bacteria Based on Cationic Cyclometalated Iridium(III) Complexes with Aggregation Induced Emission Properties

Po-Yu Ho, Sin-Ying Lee, Chuen Kam, Junfei Zhu, Guo-Gang Shan, Yuning Hong, Wai-Yeung Wong, and Sijie Chen**

Supporting Information

Fluorescence Imaging and Photodynamic Inactivation of Bacteria Based on Cationic Cyclometalated Iridium(III) Complexes with Aggregation Induced Emission Properties

Po-Yu Ho†*, Sin-Ying Lee*†*, Chuen Kam, Junfei Zhu, Guo-Gang Shan, Yuning Hong, Wai-Yeung Wong,* and Sijie Chen**

Dr. P.-Y. Ho, S.-Y. Lee, Dr. C. Kam, Dr. J. Zhu, Dr. S. Chen
Ming Wai Lau Centre of Reparative Medicine
Karolinska Institutet
Hong Kong, PR China
E-mail: sijie.chen@ki.se

Prof. W.-Y. Wong
Department of Applied Biology and Chemical Technology
The Hong Kong Polytechnic University
Hung Hom, Hong Kong, PR China
E-mail: wai-yeung.wong@polyu.edu.hk

Dr. Y. Hong
Department of Chemistry and Physics
La Trobe Institute for Molecular Science
La Trobe University
Melbourne, Victoria 3086, Australia

Dr. G.-G. Shan
Institute of Functional Materials Chemistry and National & Local United Engineering Lab for Power Battery
Faculty of Chemistry
Northeast Normal University
Changchun 130024, PR China

†These two authors contributed equally to this work.

ORCID
Po-Yu Ho: 0000-0002-2543-9541
Sin-Ying Lee: 0000-0002-8819-5871
Chuen Kam: 0000-0001-8913-167X
Guo-Gang Shan: 0000-0002-1050-219X
Yuning Hong: 0000-0002-8085-1651
Wai-Yeung Wong: 0000-0002-9949-7525
Sijie Chen: 0000-0001-5263-9189

Content

1.	Experimental section	S2
2.	Synthetic procedures of compounds	S9
3.	Molecular structures of other key chemicals in this study	S13
4.	^{1}H NMR and ^{13}C NMR spectra	S14
5.	High resolution MALDI-TOF-MS spectra	S17
6.	Crystal data and structure refinements	S19
7.	Selected structural bond lengths (Å) and angles (°) of Ir(III) complexes	S22
8.	ROS and singlet oxygen quantum yield determination results	S23
9.	Detailed quantitative analysis of colony formation	S26
10.	Theoretical calculations	S27
11.	Solid-state emission spectra and emission lifetimes of the Ir(III) complexes	S28
	References	S29

1. Experimental section

Materials and reagents

All chemical reactions were performed under an inert nitrogen atmosphere with the use of a Schlenk line. Glassware was dried in oven prior to use. Commercially available reagents were used without purification. All the reagents for chemical synthesis were purchased from Tokyo Chemical Industry Co., Ltd (TCI), Sigma-Aldrich, Acros Organics and J&K Scientific. Dry solvents were purchased from the abovementioned companies and stored in the presence of activated 3 A˚ molecular sieves. LB agar and LB broth were purchased from Invitrogen. 2′,7′-Dichlorofluorescin diacetate (DCFH-DA) and ampicillin sodium salt were purchased from

Sigma-Aldrich. Kanamycin sulfate was purchased from Thermo Fisher Scientific. Tris(2,2'-bipyridyl)ruthenium(ii) chloride hexahydrate and 1,3-diphenylisobenzofuran (DPBF) were obtained from J&K Scientific. All the reactions were monitored by thin-layer chromatography (TLC) with Merck pre-coated aluminium plates. Products were purified by column chromatography on silica gel (230−400 mesh from Merck or Davisil® LC60Å 40-63μm from W. R. Grace & Co.-Conn).

Instrumentation for chemical characterizations and photophysical measurements

Proton and carbon NMR spectra were measured in $CDCl_3$ or acetonitrile-d_3 on a Bruker AVANCE III 400 (or 500) MHz FT-NMR Spectrometer and tetramethylsilane (TMS) was exploited as an internal standard for calibrating the chemical shift. Matrix-assisted laser desorption ionization time-of-flight (MALDI-TOF) mass spectrometry was performed on an Autoflex Bruker MALDI-TOF system. UV/Vis absorption spectroscopy was performed on a Molecular Devices SpectraMax M2^{e} in different solutions at 293 K. The solution emission spectra of Ir(III) complexes were measured on a PerkinElmer Fluorescence Spectrometer LS 55.

X-ray diffraction studies

X-ray diffraction data were collected at 296 or 300 K using graphite-monochromated Mo−Kα radiation (λ = 0.71073 Å) on a Bruker D8 Venture X-Ray Diffractometer (or Bruker APEX II DUO diffractometer). The collected frames were processed with the software SAINT and an absorption correction (SADABS) was applied to the collected reflections. The structure was solved by the direct methods (SHELXTL) in conjunction with standard difference Fourier techniques and subsequently refined by full-matrix least-squares analyses on F^2.

CCDC 2064025 (**Ir(ppy)$_2$bP**), 2064026 (**Ir(1-pq)$_2$bP**) and 2064027 (**Ir(2-pq)$_2$bP**) contain(s) the supplementary crystallographic data for this paper. These data are provided free of charge by the joint Cambridge Crystallographic Data Centre and Fachinformationszentrum Karlsruhe Access Structures service www.ccdc.cam.ac.uk/structures.

Photoluminescence quantum yield (PLQY) measurement

PLQY (Φ) of the Ir(III) complexes in different solvents were determined with reference method. $[Ru(bpy)_3]^{2+}$ (Φ = 0.06 in acetonitrile) was used as the reference standard. The concentration of solution(s) was 10.0 µM or below. The quantum yield was calculated according to the equation shown below

$$\Phi_s = \Phi_r \left(\frac{A_r\ (\lambda_r)}{A_s\ (\lambda_s)}\right) \left(\frac{I\ (\lambda_r)}{I\ (\lambda_s)}\right) \left(\frac{n_s}{n_r}\right)^2 \left(\frac{\int F_s}{\int F_r}\right)$$

where, Φ is the PLQY, n is the refractive index of solvent(s), $I(\lambda)$ is the relative intensity of the exciting light at wavelength λ, $A(\lambda)$ is the absorbance of the solution at the excitation wavelength, and $\int F$ represents the integrated area under the emission spectrum. Subscripts s and r refer to the sample and references solutions, respectively.

Solid-state emission spectra and emission lifetime measurements

The iridium(III) complex(es) (1 mM in tetrahydrofuran) was spin-coated (200 rpm for 10 s, then 1000 rpm for 60 s) on a pre-cleaned quartz plate(s) (1.5 cm × 1.5 cm × 1 mm) to form a pristine thin-film, respectively.

The solid-state emission spectra and time-resolved single photon counting (TRSPC) spectra of the metal complexes were measured on a FLS1000 Photoluminescence Spectrometer at 293 K, respectively. The emission decay curves were analysed using an algorithm fitting

routine supplied by Fluoracle and were shown to follow a biexponential function in each case according to $I = I_0 + A\exp(-t \cdot \tau^{-1})$.

Sample preparation for bacteria-related experiments and singlet oxygen quantum yields determination

Stock solutions of Ir(III) complexes in DMSO with a concentration of 10 mM were prepared and stored in the 4 °C fridge prior use. The stock solutions were directly diluted to lower concentrations to make up aqueous solutions (≥ 99.9% of water) for the experiments.

Bacterial culture

Gram-negative bacteria, *E. coli* K12 and gram-positive bacteria, *S. epidermidis* were employed in this study. Both bacteria were cultured in LB medium at 37 °C for 16 hours. The concentrations of bacteria were determined by measuring the optical density at 600 nm (OD_{600}) and a specific colony forming unit (CFU) of bacteria was harvested and washed with Milli-Q water by centrifuging at 8000 rcf for 3 min. The obtained bacteria were then used for labelling, ROS generation and bacterial killing experiments.

Confocal laser scanning microscopy (CLSM)

10^9 CFU/mL of both bacterial strains were harvested and resuspended in 1 mL Milli-Q water containing 10 μM of the Ir(III) complexes respectively. After incubating at room temperature for 30 min, cells were then collected and washed with 1 mL Milli-Q water at 8000 rcf for 3 mins. The stained bacteria were then resuspended in 1 mL of Milli-Q water containing 1 μg/ml of Hoechst 33342 for 30 mins. 1.5 μL of bacterial solution was transferred to a glass slide covered

by a coverslip for imaging. Confocal imaging was performed using a Zeiss LSM 880 laser scanning confocal microscope equipped with a Plan-Apochromat 63x/1.4 NA oil objective lens, a photo-multiplier tube and a Gallium arsenide phosphide detector driven by the ZEN software (Carl Zeiss). For all three Ir(III) complexes, 405 nm laser and 550–700 nm band-pass emission filter were used. For Hoechst 33342, 405 nm laser and 410–500 nm band-pass emission filter were used. Digital images were captured with ZEN software (ZEN 2.5 lite) in grayscale and pseudo-coloured. Image processing was accomplished using ImageJ.

ROS generation detection

Bacteria, 10 μM Ir(III) complexes and 1 μM DCFH were sequentially added into corresponding wells of a 96-well plate, well mixed before adding the second/third component, and topped up to 200 μL with Milli-Q water. The plate was irradiated with white light for 1 hr. The fluorescence intensity was recorded once every 5 mins during the first half an hour and every 10 mins for the remaining time using SpectraMax M2^{e} microplate reader with an excitation and emission wavelength at 488 nm and 530 nm. The relative PL intensity (I / I_0) at 530 nm versus the irradiation time was plotted to evaluate the ROS generation.

Determination of $^1O_2$ generation quantum yield

An air-equilibrated CH_3OH solution (3 mL) containing the Ir(III) complexes and 1,3-diphenylisobenzofuran (DPBF) was introduced to a quartz cuvette of 1 cm path length and irradiated at λ = 365 nm. $Ru(bpy)_3^{2+}$ was used as a reference for $^1O_2$ sensitization (Φ_Δ = 0.70).[1] The absorbance of the complex and $Ru(bpy)_3^{2+}$ at λ = 365 nm was ca. 0.03. The absorbance of

DPBF at λ = 410 nm was monitored every 10 s. The Φ_Δ values of the complexes were determined using the following equation:

$$\Phi_\Delta^{unk} = \Phi_\Delta^{ref}\ x\ \frac{m^{unk}}{m^{ref}}\ x\ \frac{F^{ref}}{F^{unk}}$$

where m is the slope of a linear fit of the change in absorbance of DPBF at λ = 410 nm against the irradiation time, and F is the absorption correlation factor, which is given as $F = 1 - 10^{-AL}$ (A = absorbance at λ = 365 nm and L = path length of the cuvette).

In vitro bacterial photodynamic inactivation

100 μL Milli-Q water was dispensed into wells of a 96-well plate. 100 μL of 2X the top desired concentration of the Ir(III) complexes were then added into the wells in column 1. 100 μL of solutions in wells in column 1 were withdrawn and added to column 2 to make a twofold dilution. 5 μL of 10^6 CFU/mL of both bacterial strains were dispensed into the wells. The phototoxicity of Ir(III) complexes were determined by irradiating under white light with a power of 134 mW/cm^2 or keeping in the dark for 1 hr. To determine the time required for the inhibitory effect of Ir(III) complexes to bacteria, 10 μL of bacterial solutions were taken from wells at 0, 30 and 60 mins and directly diluted 10 times in Milli-Q water. 50 μL of diluted solutions were then spread on the LB agar plates and the plates were incubated at 37 °C overnight to form colonies. The colonies were imaged and counted to determine the killing effect of the Ir(III) complexes.

Morphology studies on TEM Grids

Both bacterial strains in cleaned suspensions were incubated with 10 μM of **Ir(1-pq)$_2$bP**. The bacterial cells were irradiated under white light with a power of 134 mW/cm^2 or kept in the dark for 30 mins and then washed. The samples were then mixed with 2% phosphotungstic acid

hydrate with ultra-purified water for 25 mins. A copper grid was first immersed into the phosphotungstic acid hydrate solution for 2 mins. The grid was then washed 3 times with ultra-purified water for 10 seconds each. Excess liquid from the grid was wicked away using a wedge of filter paper placed at the edge of the grid. The grid was then placed in a grid box to dry overnight and stored in a desiccator until analysis. TEM analysis was performed by using JEM-2100 Electron microscope (200 KV).

Statistical analysis

Bacterial colony formation and survival rate: error bars represent the standard deviation derived from three independent measurements, and all the statistical analysis was performed using Microsoft Excel. The images were processed with Prism.

2. Synthetic procedures of compounds

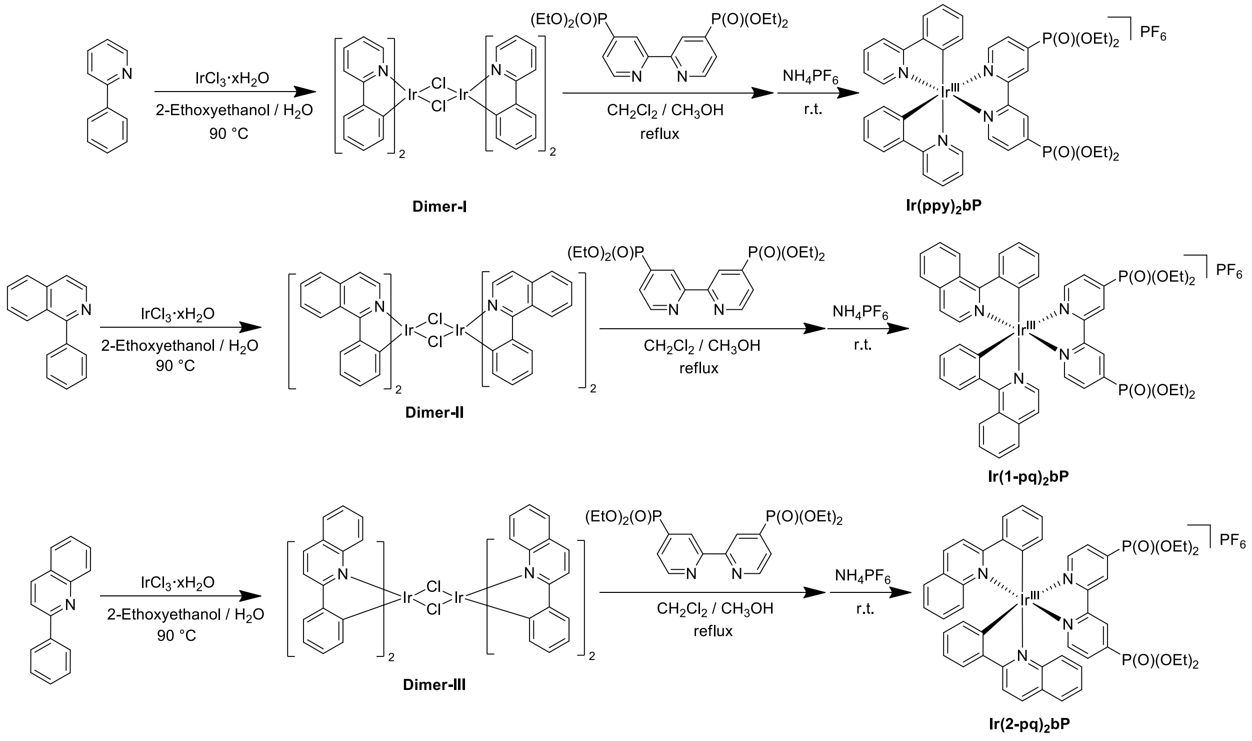

Scheme S1. Synthetic routes of the intermediate dimers and the Ir(III) complexes investigated.

Dimer-I. A mixture of 2-phenylpyridine (220 mg, 1.418 mmol) and $IrCl_3 \cdot xH_2O$ (250 mg, 0.709 mmol) in a binary solvent system (2-ethoxyethanol (4.5 mL) and water (1.5 mL)) was stirred and heated to 90 °C under nitrogen atmosphere overnight. The reaction mixture, after cooling down, a red slurry was formed. The orange participate was filtered out and washed with water and minimum amount of ethanol. The obtained solid residue after vacuum drying (220 mg, 0.205 mmol) was directly used for next reaction without further purification and characterization.

Dimer-II. A mixture of 1-phenylisoquinoline (1,164 mg, 5.672 mmol) and $IrCl_3 \cdot xH_2O$ (800 mg, 2.269 mmol) in a binary solvent system (2-ethoxyethanol (12 mL) and water (4 mL)) was stirred and heated to 90 °C under nitrogen atmosphere overnight. The reaction mixture, after cooling

down, a red slurry was formed. The red participate was filtered out and washed with water and minimum amount of ethanol. The obtained solid residue after vacuum drying (970 mg, 0.762 mmol) was directly used for next reaction without further purification and characterization.

Dimer-III. A mixture of 2-Phenylquinoline (169 mg, 0.823 mmol) and $IrCl_3{\cdot}xH_2O$ (145 mg, 0.412 mmol) in a binary solvent system (2-ethoxyethanol (4.5 mL) and water (1.5 mL)) was stirred and heated to 90 °C under nitrogen atmosphere overnight. The reaction mixture, after cooling down, a red slurry was formed. The red participate was filtered out and washed with water and minimum amount of ethanol. The obtained solid residue after vacuum drying (110 mg, 0.086 mmol) was directly used for next reaction without further purification and characterization.

Ir(ppy)$_2$bP. A mixture of **Dimer-I** (110 mg, 0.103 mmol) and tetraethyl [2,2'-bipyridine]-4,4'-diylbis(phosphonate)[2] (88 mg, 0.206 mmol) in a binary solvent system (dichloromethane (6 mL) and methanol (3 mL)) was stirred and heated to reflux under nitrogen atmosphere overnight. After cooling, ammonium hexafluorophosphate (168 mg, 1.03 mmol) was added into reaction mixture for anion exchange reaction, and the solvent was then removed under reduced pressure after 1 hr. The residue was purified by column chromatography on silica gel using a 1:20 mixture of CH_3OH and dichloromethane as eluent to give **Ir(ppy)$_2$bP** (155 mg, 0.144 mmol, 70%) as a red solid. The single crystal of **Ir(ppy)$_2$bP** for X-ray diffraction was grown by diffusion of diethyl ether into the dichloromethane solution. ^{1}H NMR (CD_3CN, 400 MHz): δ = 9.04 (d, 2H, J = 13.6 Hz, Ar), 8.18−8.11 (m, 4H, Ar), 7.88−7.74 (m, 8H, Ar), 7.01−6.91 (m, 4H, Ar), 6.83−6.80 (m, 2H, Ar), 6.20−6.18 (m, 2H, Ar), 4.11−4.04 (m, 8H, alkyl), 1.22−1.16 ppm (m, 12H, alkyl); ^{13}C NMR (125 MHz, $CDCl_3$): δ = 167.20, 155.89, 155.78, 150.67, 150.57, 149.56,

149.16, 143.56, 141.78, 140.30, 138.49, 131.67, 130.93, 130.08, 130.02, 127.19, 127.11, 124.81, 124.03, 123.08, 119.69 (Ar), 64.12, 64.08, 64.00, 63.95, 16.43, 16.38 ppm (alkyl). HRMS (MALDI-TOF, *m*/*z*): $[(M–PF_6)^+]$ 929.2217; calcd for $(C_{40}H_{42}IrN_4O_6P_2^+)$ 929.2209.

Ir(1-pq)$_2$bP. A mixture of **Dimer-II** (100 mg, 0.093 mmol) and tetraethyl [2,2'-bipyridine]-4,4'-diylbis(phosphonate) (80 mg, 0.187 mmol) in a binary solvent system (dichloromethane (8 mL) and methanol (4 mL)) was stirred and heated to reflux under nitrogen atmosphere overnight. After cooling, ammonium hexafluorophosphate (152 mg, 0.93 mmol) was added into reaction mixture for anion exchange reaction, and the solvent was then removed under reduced pressure after 1 hr. The residue was purified by column chromatography on silica gel using a 1:20 mixture of CH_3OH and dichloromethane as eluent to give **Ir(1-pq)$_2$bP** (125 mg, 0.106 mmol, 57%) as a deep red solid. The single crystal **Ir(1-pq)$_2$bP** for X-ray diffraction was grown by diffusion of diethyl ether into the dichloromethane solution.^{1}H NMR ($CDCl_3$, 500 MHz): δ = 8.90−8.89 (m, 2H, Ar), 8.82 (d, J = 13.5 Hz, 2H, Ar), 8.24 (d, 2H, J = 8 Hz, Ar), 7.92−7.83 (m, 4H, Ar), 7.79−7.70 (m, 4H, Ar), 7.70−7.68 (m, 2H, Ar), 7.50−7.44 (m, 4H, Ar), 7.12−7.09 (m, 2H, Hz, Ar), 6.91−6.88 (m, 2H, Ar), 6.20 (d, 2H, J = 7.5 Hz, Ar), 4.30−4.18 (m, 8H, alkyl), 1.40−1.36 ppm (m, 12H, alkyl); ^{13}C NMR (125 MHz, $CDCl_3$): δ = 168.65, 155.89, 155.77, 152.37, 150.60, 150.50, 145.64, 141.74, 141.09, 140.26, 137.31, 132.17, 131.84, 131.06, 130.73, 130.03, 129.97, 128.79, 127.85, 127.41, 127.33, 126.93, 126.33, 122.77, 122.61 (Ar), 64.12, 64.07, 63.99, 63.95, 16.43, 16.38 ppm (alkyl). HRMS (MALDI-TOF, *m*/*z*): $[(M–PF_6)^+]$ 1029.3071; calcd for $(C_{48}H_{46}IrN_4O_6P_2^+)$ 1029.2522.

Ir(2-pq)$_2$bP. A mixture of **Dimer-III** (111 mg, 0.104 mmol) and tetraethyl [2,2'-bipyridine]-4,4'-diylbis(phosphonate) (89 mg, 0.207 mmol) in a binary solvent system (dichloromethane (8 mL) and methanol (4 mL)) was stirred and heated to reflux under nitrogen atmosphere overnight. After cooling, ammonium hexafluorophosphate (170 mg, 1.04 mmol) was added into reaction mixture for anion exchange reaction, and the solvent was then removed under reduced pressure after 1 hr. The residue was purified by column chromatography on silica gel using a 1:20 mixture of CH_3OH and dichloromethane as eluent to give **Ir(2-pq)$_2$bP** (86 mg, 0.073 mmol, 35%) as a deep red solid. The single crystal **Ir(2-pq)$_2$bP** for X-ray diffraction was grown by diffusion of diethyl ether into the dichloromethane solution. ^{1}H NMR (CD_3CN, 400 MHz): δ = 8.64 (d, 2H, J = 13.6 Hz, Ar), 8.44−8.41 (m, 6H, Ar), 8.15−8.13 (m, 2H, Ar), 7.84−7.79 (m, 4H, Ar), 7.34−7.25 (m, 4H, Ar), 7.09−7.01 (m, 4H, Ar), 6.75−6.72 (m, 2H, Ar), 6.45−6.43 (m, 2H, Ar), 4.00−3.93 (m, 8H, alkyl), 1.11−1.06 ppm (m, 12H, alkyl); ^{13}C NMR (125 MHz, $CDCl_3$): δ = 169.85, 155.14, 155.04, 149.27, 148.61, 148.51, 147.16, 145.24, 142.06, 140.57, 140.48, 134.58, 131.27, 131.19, 129.75, 129.68, 129.47, 127.81, 127.62, 127.01, 125.50, 125.42, 124.41, 123.77, 118.29 (Ar), 64.13, 64.08, 64.01, 63.96, 16.51, 16.46, 16.42, 16.37, 16.35, 16.31 ppm (alkyl). HRMS (MALDI-TOF, m/z): [$(M–PF_6)^+$] 1029.2528; calcd for ($C_{48}H_{46}IrN_4O_6P_2^+$) 1029.2522.

3. Molecular structures of other key chemicals in this study

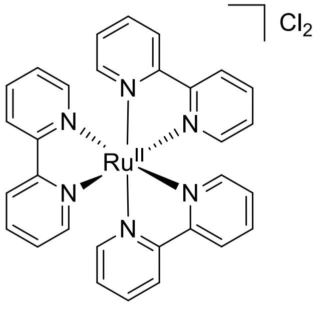

Tris(bipyridine)ruthenium(II) chloride

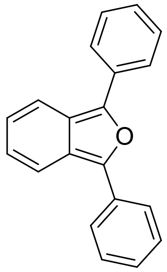

1,3-diphenylisobenzofuran

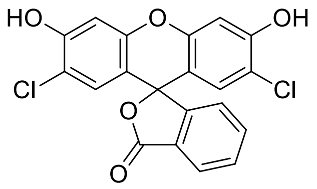

2',7'-dichlorodihydrofluorescin (DCFH)

Figure S1. Molecular structures of other key chemicals in this study.

4. ^{1}H NMR and ^{13}C NMR spectra

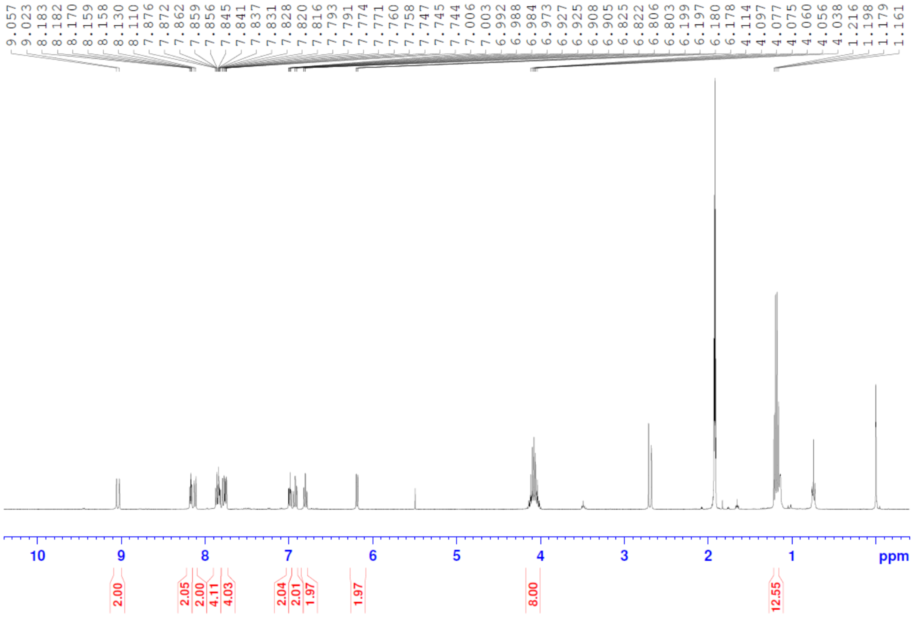

Figure S2. ^{1}H NMR of **Ir(ppy)$_2$bP** in acetonitrile-d$_3$.

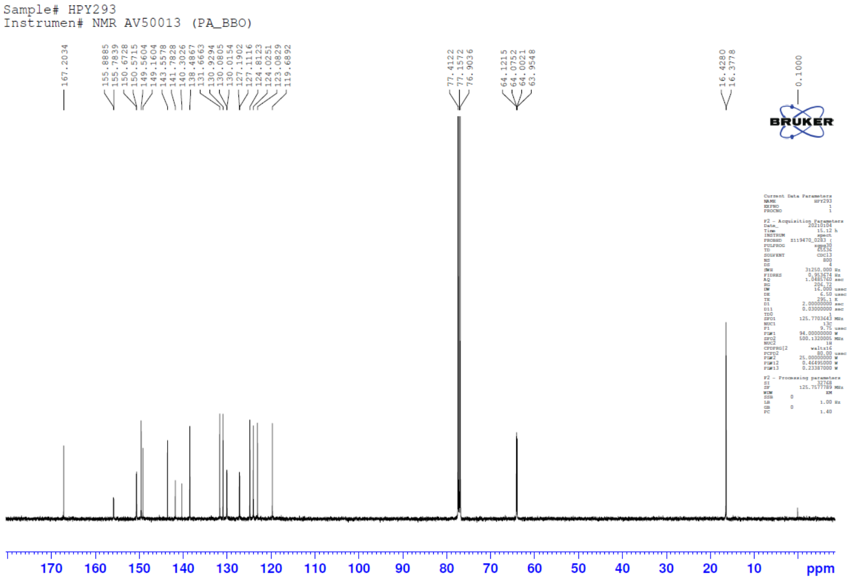

Figure S3. ^{13}C NMR of **Ir(ppy)$_2$bP** in $CDCl_3$.

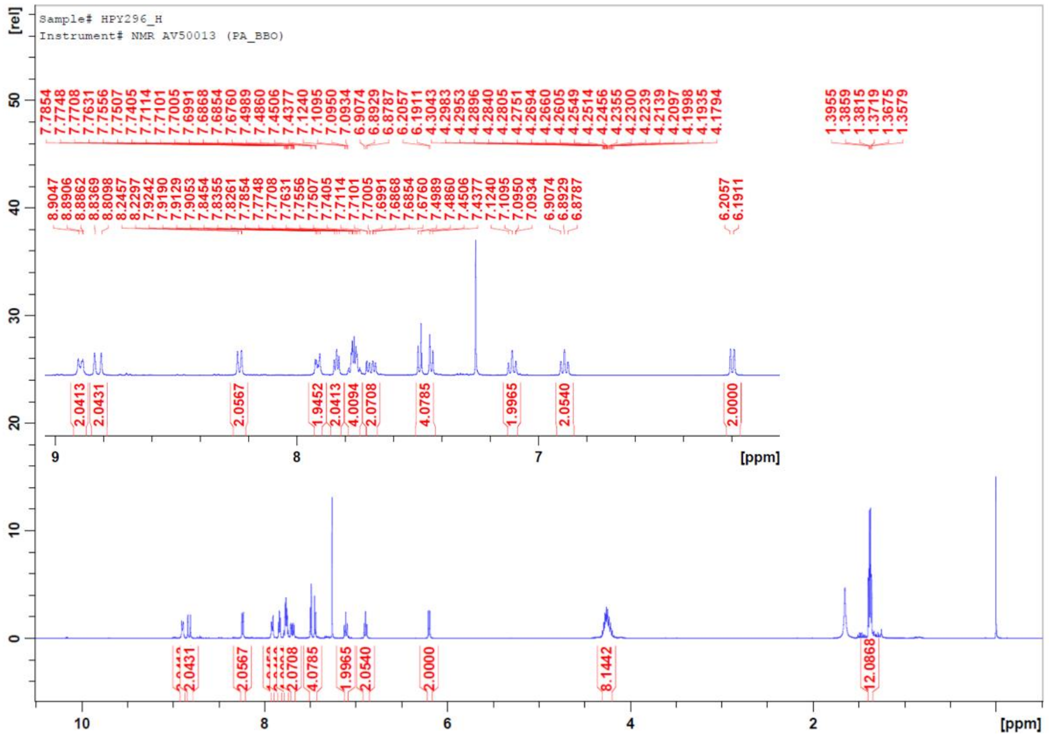

Figure S4. ^{1}H NMR of **Ir(1-pq)$_2$bP** in $CDCl_3$.

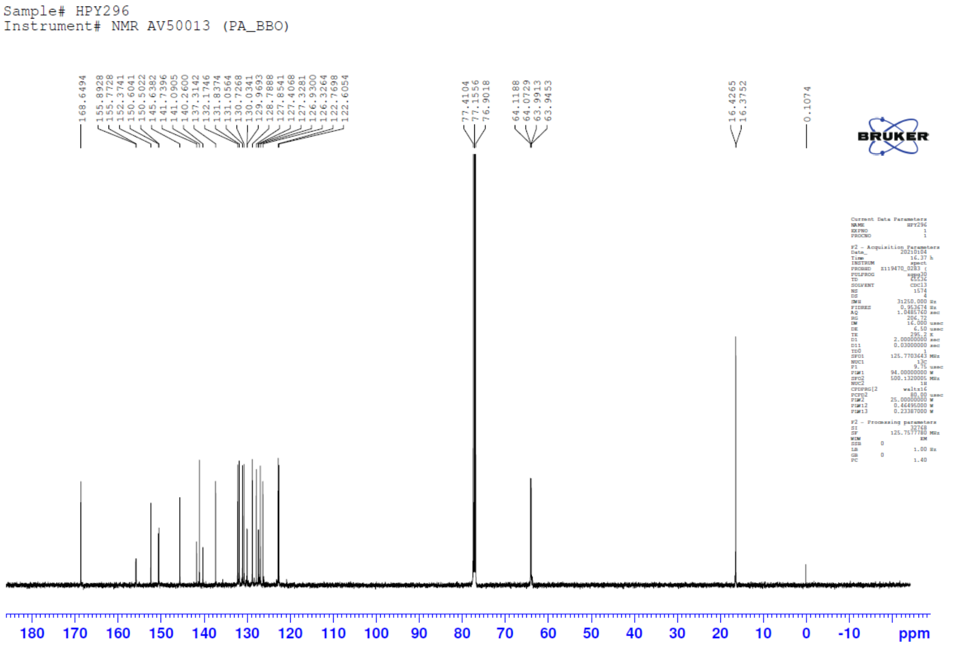

Figure S5. ^{13}C NMR of **Ir(1-pq)$_2$bP** in $CDCl_3$.

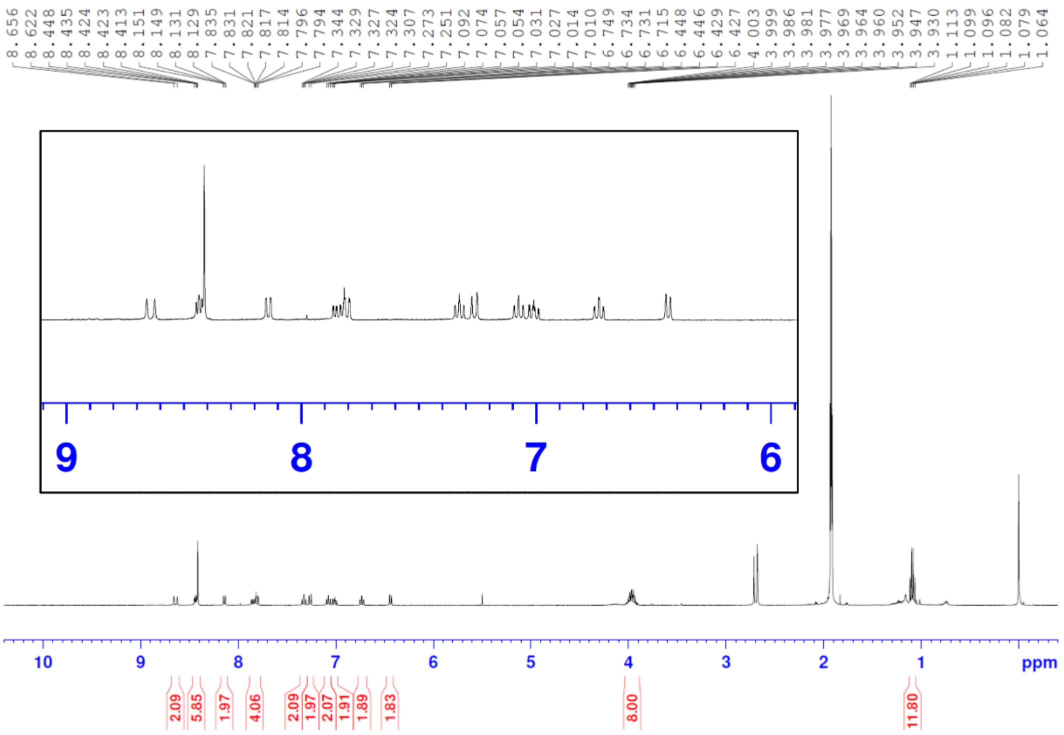

Figure S6. ^{1}H NMR of **Ir(2-pq)$_2$bP** in acetonitrile-d$_3$.

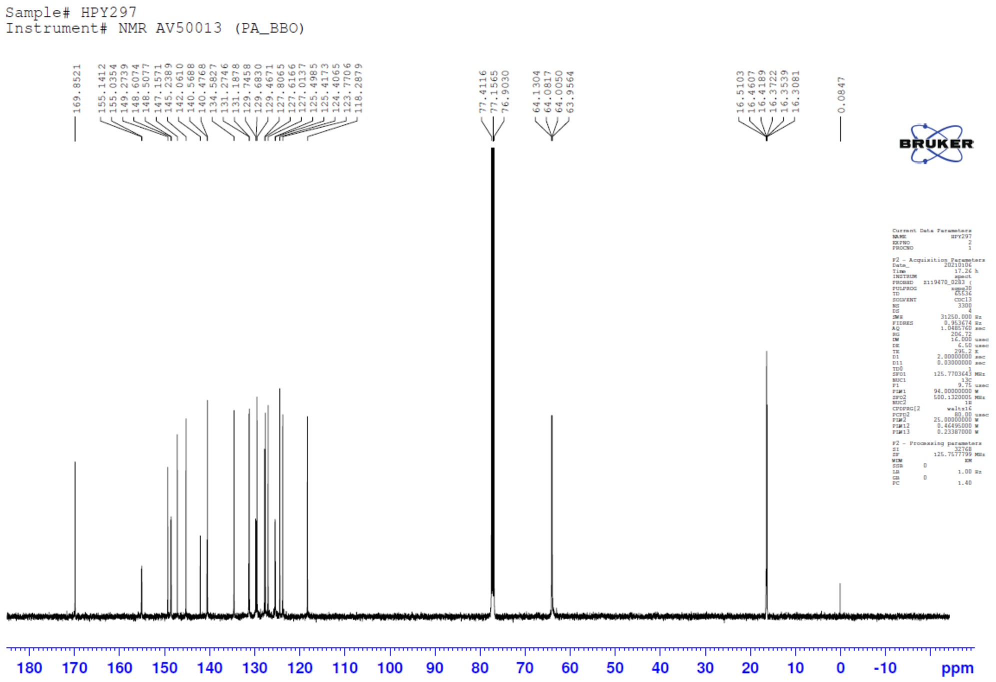

Figure S7. ^{13}C NMR of **Ir(2-pq)$_2$bP** in $CDCl_3$.

5. High resolution MALDI-TOF-MS spectra

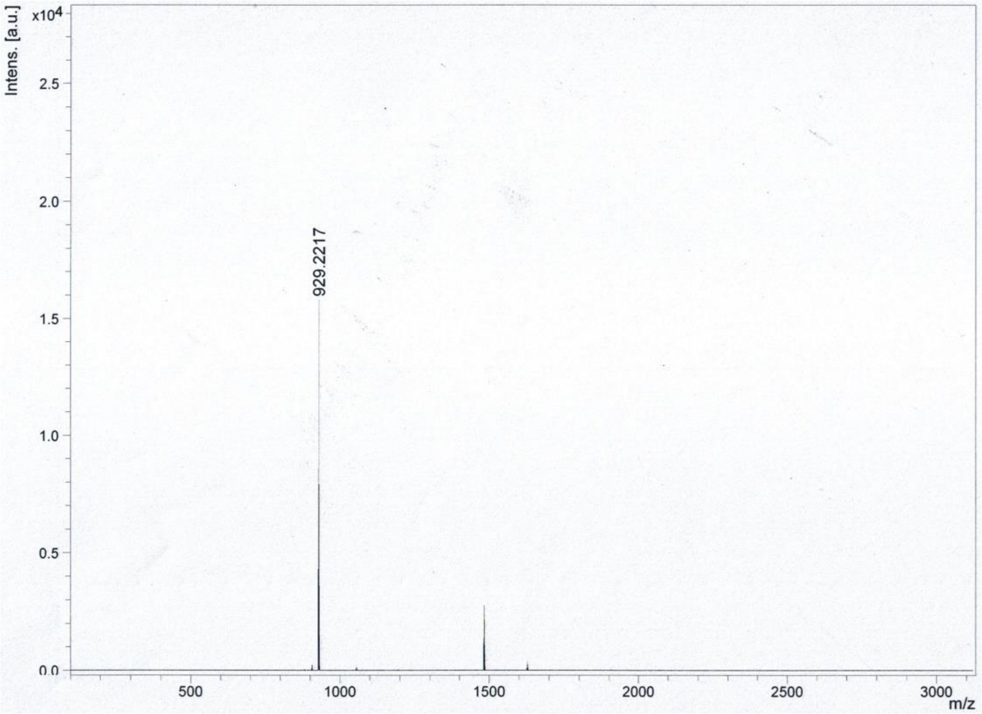

Figure S8. High resolution MALDI-TOF mass spectrum of **Ir(ppy)$_2$bP**.

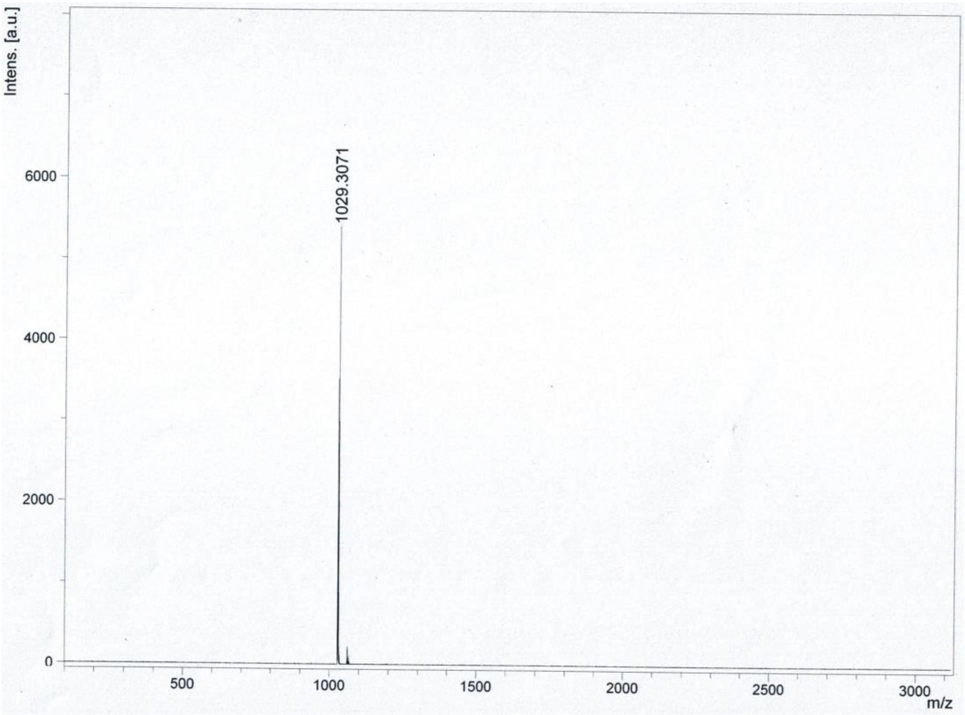

Figure S9. High resolution MALDI-TOF mass spectrum of **Ir(1-pq)$_2$bP**.

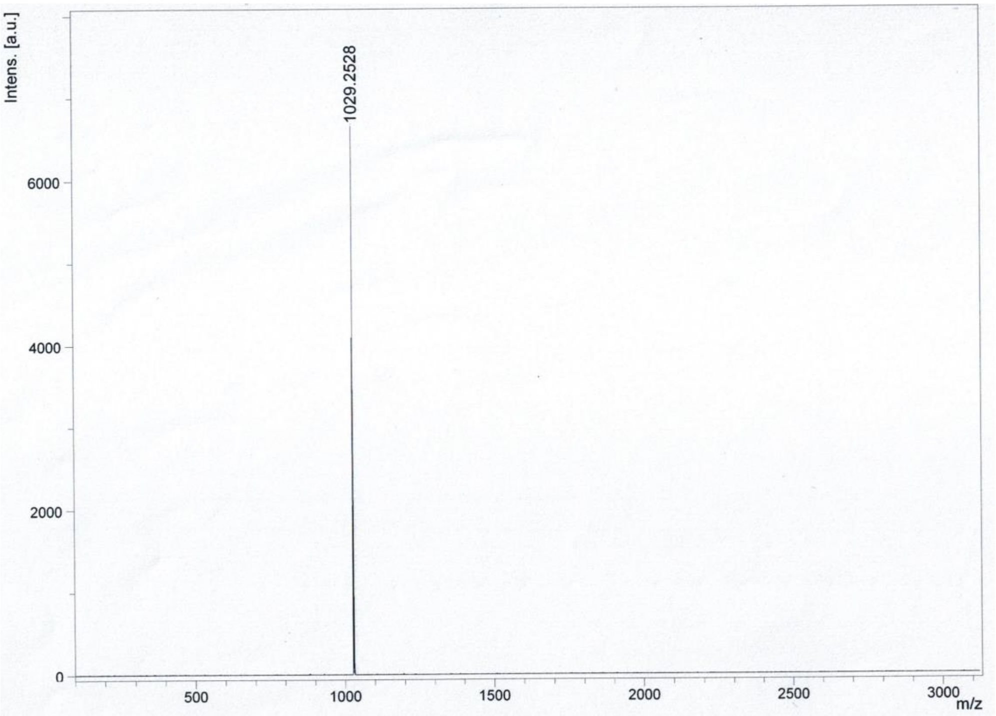

Figure S10. High resolution MALDI-TOF mass spectrum of **Ir(2-pq)$_2$bP**.

6. Crystal data and structure refinements

Table S1. Crystal data and structure refinement for **Ir(ppy)$_2$bP**.

Empirical formula	$C_{40}H_{42}F_6IrN_4O_6P_3$	
Formula weight	1073.88	
Temperature	300(2) K	
Wavelength	0.71073 Å	
Crystal system	Monoclinic	
Space group	P2$_1$/c	
Unit cell dimensions	a = 9.8848(2) Å	a= 90°.
	b = 24.6915(7) Å	b= 100.4990(10)°.
	c = 19.9029(5) Å	g = 90°.
Volume	4776.4(2) Å^3	
Z	4	
Density (calculated)	1.493 Mg/m^3	
Absorption coefficient	2.964 mm^{-1}	
F(000)	2136	
Crystal size	0.3 x 0.2 x 0.2 mm^3	
Theta range for data collection	2.252 to 25.250°.	
Index ranges	-10<=h<=11, -29<=k<=29, -23<=l<=23	
Reflections collected	80631	
Independent reflections	8627 [R(int) = 0.0418]	
Completeness to theta = 25.242°	99.8 %	
Absorption correction	multi-scan	
Max. and min. transmission	0.7456 and 0.5660	
Refinement method	Full-matrix least-squares on F^2	
Data / restraints / parameters	8627 / 78 / 545	
Goodness-of-fit on F^2	1.086	
Final R indices [I>2sigma(I)]	R_1 = 0.0430, wR_2 = 0.1265	
R indices (all data)	R_1 = 0.0513, wR_2 = 0.1319	
Extinction coefficient	n/a	
Largest diff. peak and hole	0.958 and -1.500 e.Å^{-3}	

Table S2. Crystal data and structure refinement for **Ir(1-pq)$_2$bP**.

Empirical formula	$C_{48}H_{46}F_6IrN_4O_{6.5}P_3$	
Formula weight	1182.00	
Temperature	300(2) K	
Wavelength	0.71073 Å	
Crystal system	Triclinic	
Space group	P-1	
Unit cell dimensions	a = 11.9376(6) Å	a= 89.502(2)°.
	b = 20.2295(9) Å	b= 85.703(2)°.
	c = 21.7406(10) Å	g = 76.356(2)°.
Volume	5087.5(4) Å^3	
Z	4	
Density (calculated)	1.543 Mg/m^3	
Absorption coefficient	2.792 mm^{-1}	
F(000)	2360	
Crystal size	0.3 x 0.2 x 0.2 mm^3	
Theta range for data collection	2.244 to 25.250°.	
Index ranges	-14<=h<=14, -24<=k<=24, -26<=l<=26	
Reflections collected	123040	
Independent reflections	18425 [R(int) = 0.0775]	
Completeness to theta = 25.242°	99.9 %	
Absorption correction	multi-scan	
Max. and min. transmission	0.7456 and 0.5998	
Refinement method	Full-matrix least-squares on F^2	
Data / restraints / parameters	18425 / 200 / 1242	
Goodness-of-fit on F^2	1.051	
Final R indices [I>2sigma(I)]	R_1 = 0.0449, wR_2 = 0.1000	
R indices (all data)	R_1 = 0.0734, wR_2 = 0.1105	
Extinction coefficient	n/a	
Largest diff. peak and hole	0.765 and -0.961 e.Å^{-3}	

Table S3. Crystal data and structure refinement for **Ir(2-pq)$_2$bP**.

Empirical formula	$C_{97}H_{94}Cl_2F_{12}Ir_2N_8O_{12}P_6$	
Formula weight	2432.92	
Temperature	296.15 K	
Wavelength	0.71073 Å	
Crystal system	Triclinic	
Space group	P-1	
Unit cell dimensions	a = 13.5560(11) Å	a= 96.864(2)°.
	b = 14.1112(11) Å	b= 104.126(2)°.
	c = 15.6859(13) Å	g = 112.904(2)°.
Volume	2602.0(4) Å^3	
Z	1	
Density (calculated)	1.553 Mg/m^3	
Absorption coefficient	2.781 mm^{-1}	
F(000)	1214.0	
Crystal size	0.4 x 0.3 x 0.2 mm^3	
Theta range for data collection	2.758 to 52.746°.	
Index ranges	-16<=h<=16, -17<=k<=17, -19<=l<=19	
Reflections collected	53612	
Independent reflections	10602 [R(int) = 0.0399]	
Data / restraints / parameters	10602 / 0 / 644	
Goodness-of-fit on F^2	1.100	
Final R indices [I>2sigma(I)]	R_1 = 0.0343, wR_2 = 0.1004	
R indices (all data)	R_1 = 0.0427, wR_2 = 0.1066	
Largest diff. peak and hole	1.52 and -0.56 e.Å^{-3}	

7. Selected structural bond lengths (Å) and angles (°) of Ir(III) complexes

Table S4. Selected structural bond length (Å) and angles (°) in complex **Ir(ppy)$_2$bP**.

Bond Distances (Å)			
Ir(1)−C(11)	2.009(6)	Ir(1)−N(4)	2.055(5)
Ir(1)−C(12)	2.030(6)	Ir(1)−N(1)	2.140(4)
Ir(1)−N(3)	2.046(5)	Ir(1)−N(2)	2.147(5)
Bond Angles (°)			
N(3)−Ir(1)−N(4)	173.66(18)	N(3)−Ir(1)−N(1)	97.10(18)
C(11)−Ir(1)−N(1)	174.1(2)	N(3)−Ir(1)−N(2)	86.83(17)
C(12)−Ir(1)−N(2)	172.40(19)	N(1)−Ir(1)−N(2)	76.29(16)
C(11)−Ir(1)−N(3)	80.0(2)	C(11)−Ir(1)−C(12)	89.2(2)
C(12)−Ir(1)−N(3)	95.3(2)		

Table S5. Selected structural bond length (Å) and angles (°) in complex **Ir(1-pq)$_2$bP**.

Bond Distances (Å)			
Ir(1)−C(15)	2.008(5)	Ir(1)−N(2)	2.147(5)
Ir(1)−C(16)	1.999(6)	Ir(1)−N(3)	2.148(4)
Ir(1)−N(1)	2.045(5)	Ir(1)−N(4)	2.035(5)
Bond Angles (°)			
C(15)−Ir(1)−N(3)	172.4(2)	C(15)−Ir(1)−N(4)	93.9(2)
C(16)−Ir(1)−N(2)	172.63(19)	C(15)−Ir(1)−C(16)	90.3(2)
N(4)−Ir(1)−N(1)	170.53(18)	N(1)−Ir(1)−N(2)	88.12(18)
C(15)−Ir(1)−N(1)	79.4(2)	C(16)−Ir(1)−N(4)	79.2(2)
C(15)−Ir(1)−N(2)	97.0(2)		

Table S6. Selected structural bond length (Å) and angles (°) in complex **Ir(2-pq)$_2$bP**.

Bond Distances (Å)			
Ir(1)−C(15)	2.006(4)	Ir(1)−N(2)	2.093(4)
Ir(1)−C(30)	2.010(5)	Ir(1)−N(3)	2.173(4)
Ir(1)−N(1)	2.083(4)	Ir(1)−N(4)	2.178(3)
Bond Angles (°)			
C(15)−Ir(1)−N(4)	169.37(15)	N(1)−Ir(1)−N(3)	84.67(14)
C(30)−Ir(1)−N(3)	172.55(15)	N(1)−Ir(1)−N(4)	103.18(14)
N(1)−Ir(1)−N(2)	172.13(15)	N(3)−Ir(1)−N(4)	75.04(13)
C(15)−Ir(1)−N(1)	79.73(18)	C(15)−Ir(1)−C(30)	91.30(18)
C(30)−Ir(1)−N(1)	92.96(19)		

8. ROS and singlet oxygen quantum yield determination results

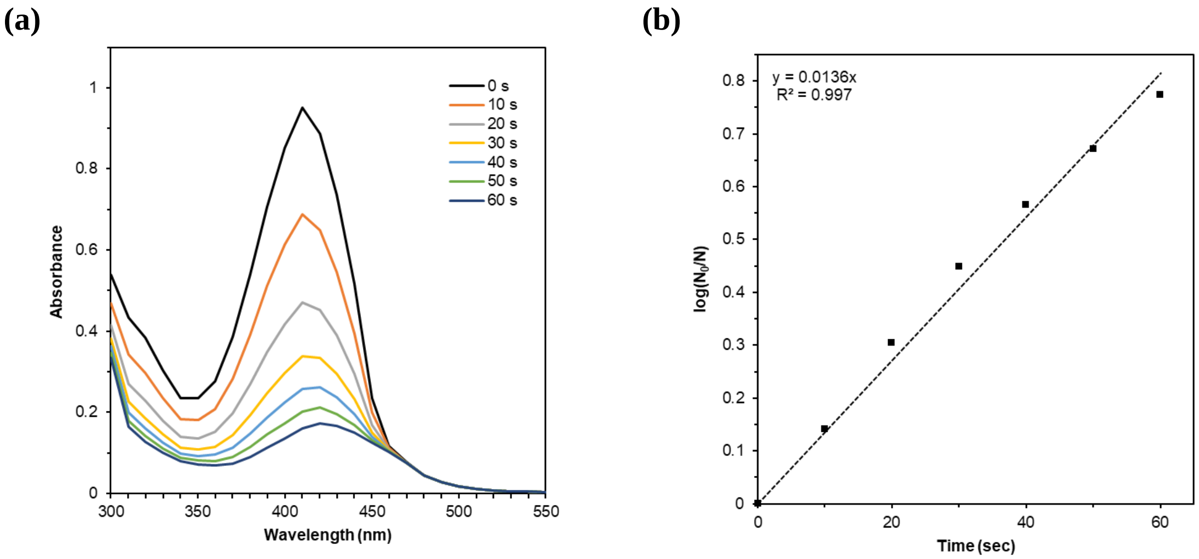

Figure S11. a) UV-vis absorbance spectra of CH_3OH solution containing $Ru(bpy)_3Cl_2$ and DPBF upon irradiation of 365 nm in every 10-seconds interval. b) Time dependence of DPBF concentration ratio based on the absorbance maximum at ca. 410 nm upon irradiation of 365 nm in the presence of $Ru(bpy)_3Cl_2$.

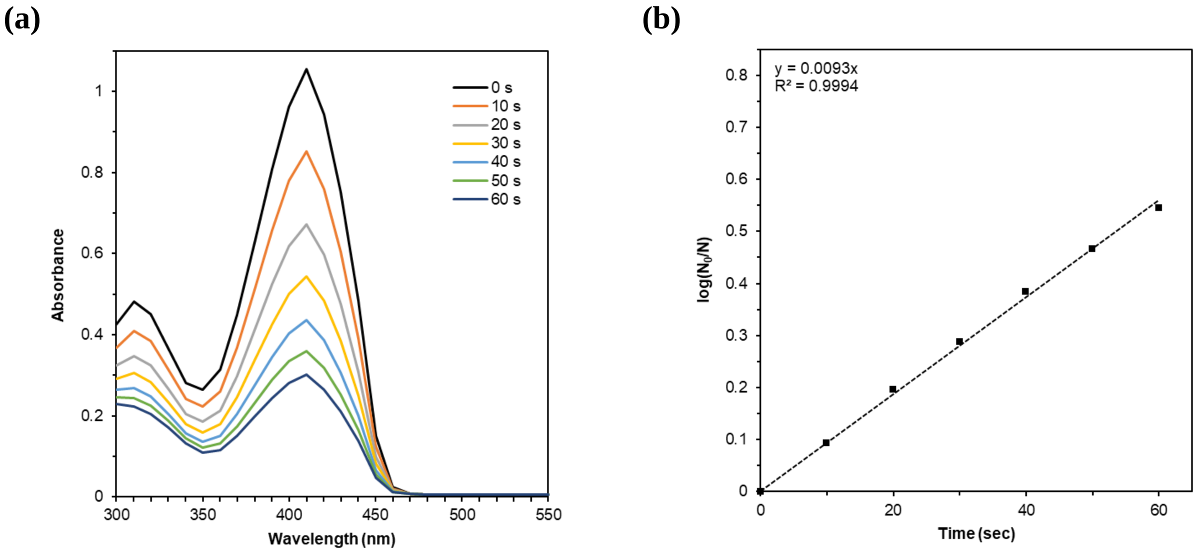

Figure S12. a) UV-vis absorbance spectra of CH_3OH solution containing $\mathbf{Ir(ppy)_2bP}$ and DPBF upon irradiation of 365 nm in every 10-seconds interval. b) Time dependence of DPBF concentration ratio based on the absorbance maximum at ca. 410 nm upon irradiation of 365 nm in the presence of $\mathbf{Ir(ppy)_2bP}$.

(a) (b)

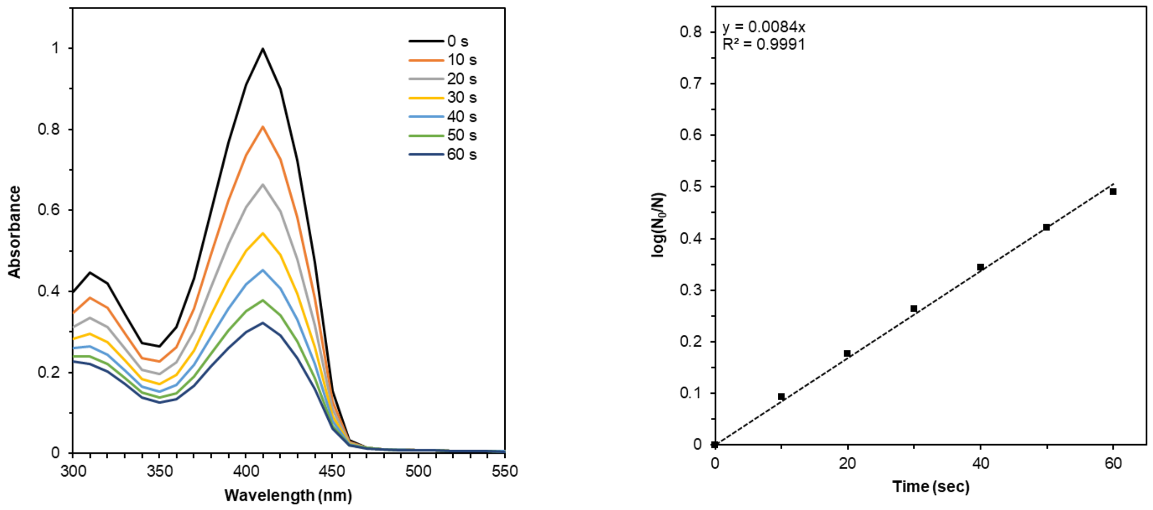

Figure S13. a) UV-vis absorbance spectra of CH_3OH solution containing **Ir(1-pq)$_2$bP** and DPBF upon irradiation of 365 nm in every 10-seconds interval. b) Time dependence of DPBF concentration ratio based on the absorbance maximum at ca. 410 nm upon irradiation of 365 nm in the presence of **Ir(1-pq)$_2$bP**.

(a) (b)

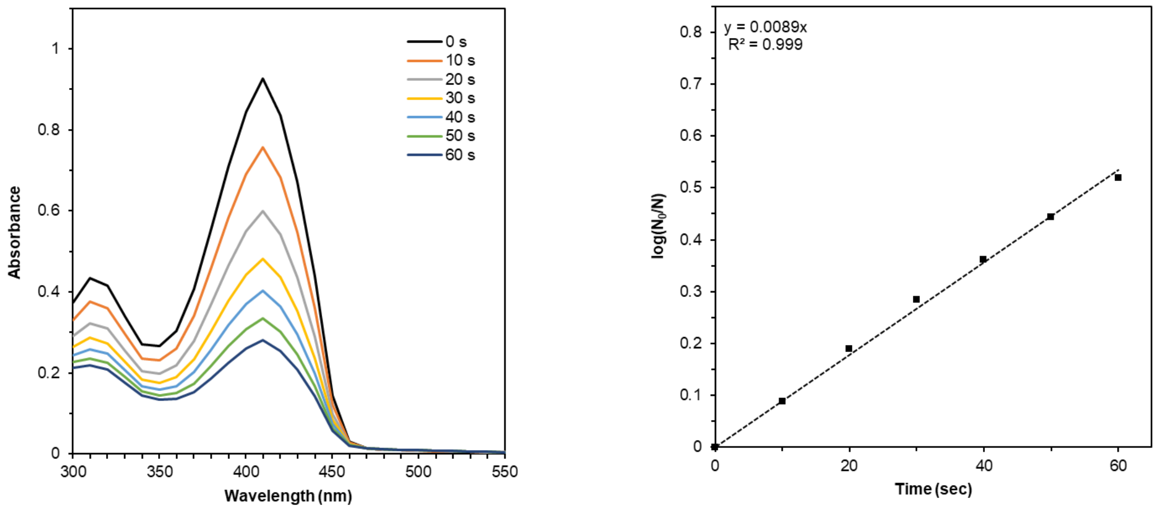

Figure S14. a) UV-vis absorbance spectra of CH_3OH solution containing **Ir(2-pq)$_2$bP** and DPBF upon irradiation of 365 nm in every 10-seconds interval. b) Time dependence of DPBF concentration ratio based on the absorbance maximum at ca. 410 nm upon irradiation of 365 nm in the presence of **Ir(2-pq)$_2$bP**.

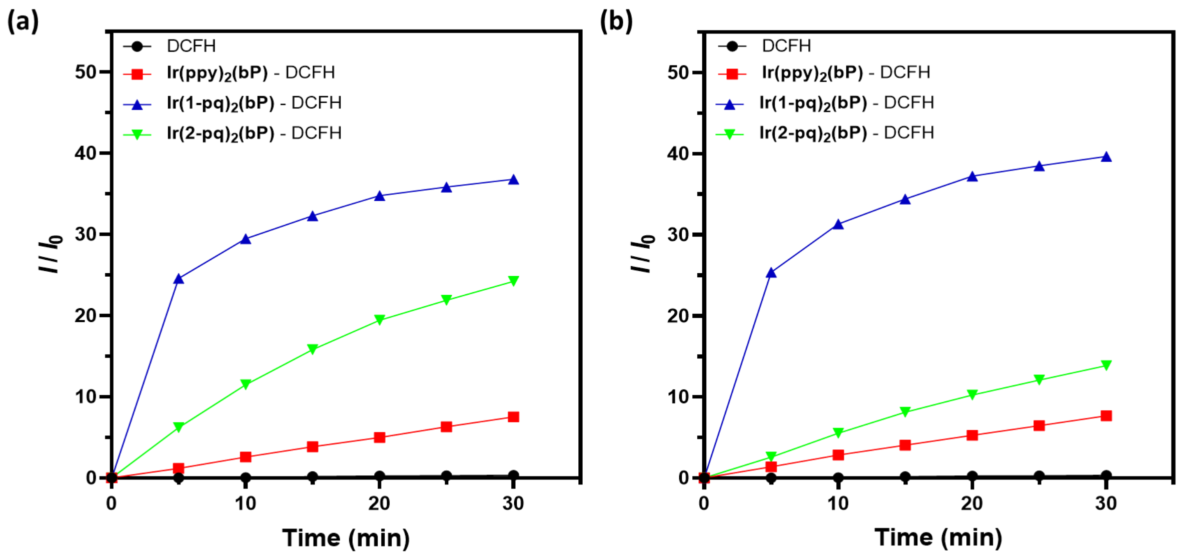

Figure S15. PL (excitation at 488 nm and emission at 530 nm) intensity ratio of the solution mixtures of 1 μM 2′,7′-dichlorodihydrofluorescin (DCFH only (●)) and additional 10 μM Ir(III) complex (**Ir(ppy)$_2$bP** (■), **Ir(1-pq)$_2$bP** (▲) and **Ir(1-pq)$_2$bP** (▼)) in the presence of a) *E. Coli* K12 and b) *S. epidermidis* (~ 10^6 CFU/ml) upon white light irradiation over time.

9. **Detailed quantitative analysis of colony formation**

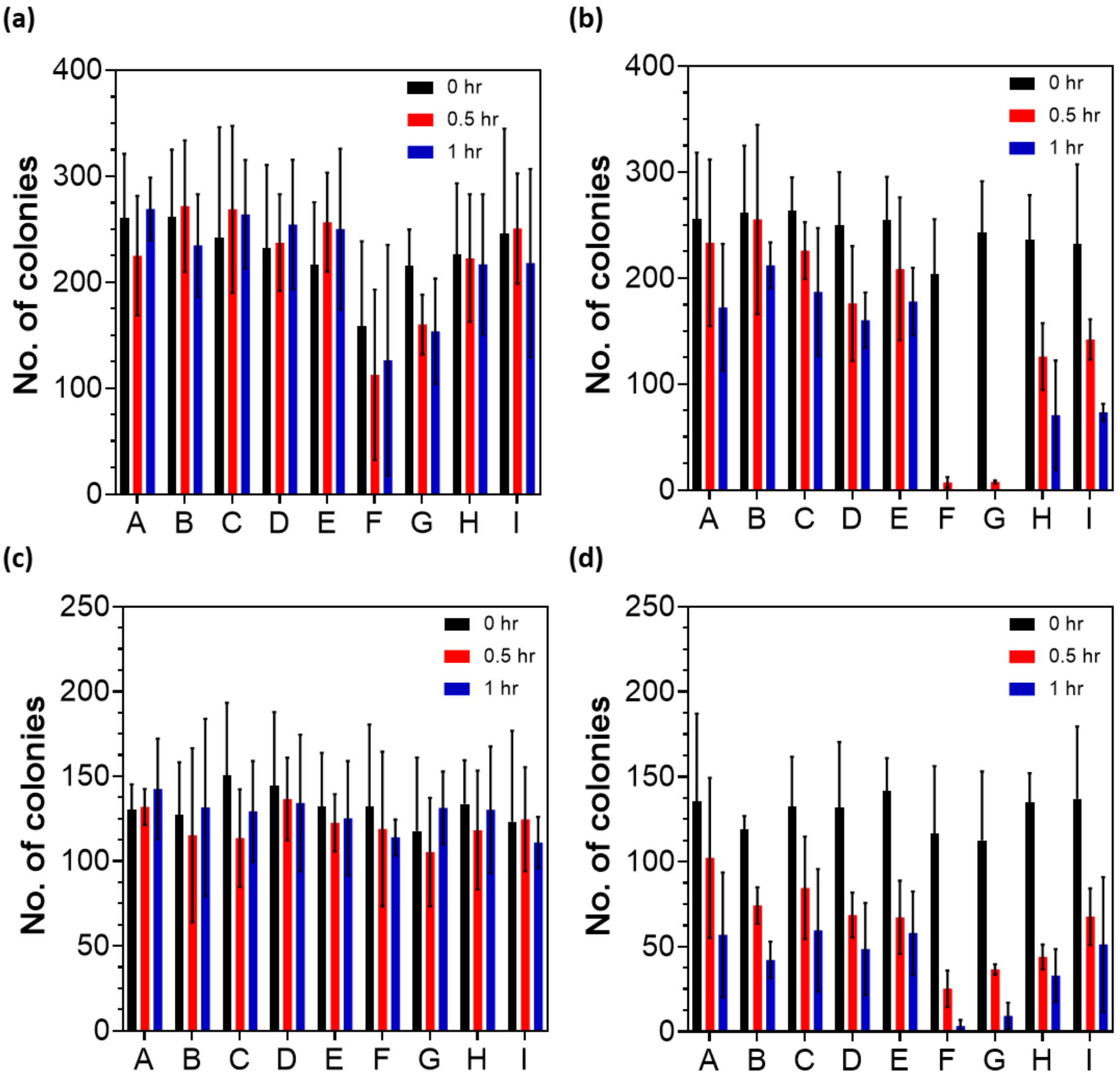

Figure S16. Plate-count results of a,b) *E. coli* K-12 and c,d) *S. epidermidis* treated with the blank control (BC), ampicillin (Amp), or Ir(III) complexes (5 or 10 μM) in darkness or upon white light illumination (LED with a power of 134 mW/cm^2) for different durations. Conditions: (A) BC, (B) Amp at 10 μM, (C) Amp at 5 μM, (D) **Ir(ppy)$_2$bP** at 10 μM, (E) **Ir(ppy)$_2$bP** at 5 μM, (F) **Ir(1-pq)$_2$bP** at 10 μM, (G) **Ir(1-pq)$_2$bP** at 5 μM, (H) **Ir(2-pq)$_2$bP** at 10 μM and (I) **Ir(2-pq)$_2$bP** at 5 μM.

10. Theoretical calculations

Geometry optimizations were performed for the singlet ground state (S_0), the lowest emitting triple excited state (T_1), the lowest ^{3}MC excited state and the transition states for $T_1 \rightarrow {}^3$MC conversions using density functional theory (DFT)[3] at the hybrid functional B3LYP level within the Gaussian 09 software package.[4] The 6-31G* basis sets for C, H, N atoms and the LANL2DZ for Ir atom were selected for the geometry optimization. An effective core potential (ECP) replaces the inner core electrons of iridium leaving the outer core $(5s)^2(5p)^6$ electrons and the $(5d)^6$ valence electrons of Ir(III). The nature of the stationary points was confirmed by computing the Hessian at the same level of theory. The minimum energy crossing point (MECP) between the S_0 and the ^{3}MC potential energy surface was optimized using Harvey's algorithm.[5]

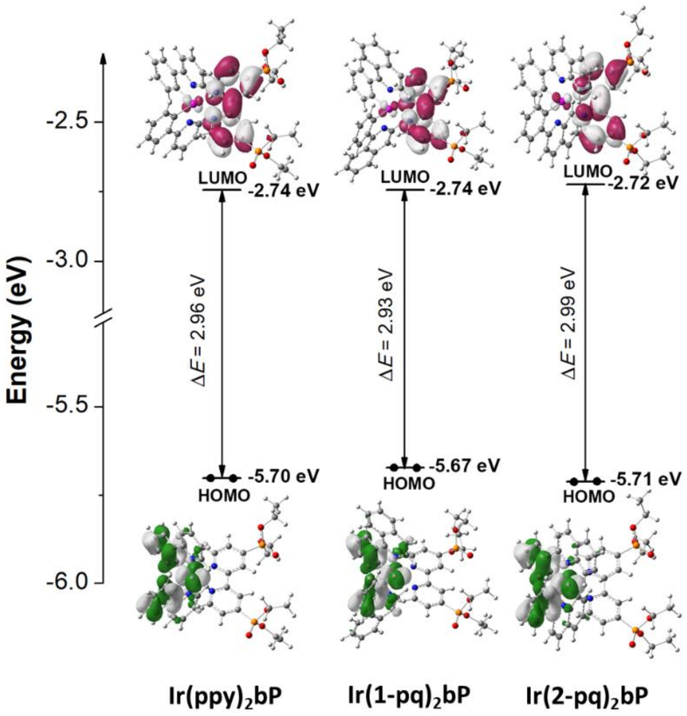

Figure S17. Electronic density contours of HOMOs and LUMOs for **Ir(ppy)$_2$bP**, **Ir(1-pq)$_2$bP** and **Ir(1-pq)$_2$bP**, respectively.

11. Solid-state emission spectra and emission lifetimes of the Ir(III) complexes

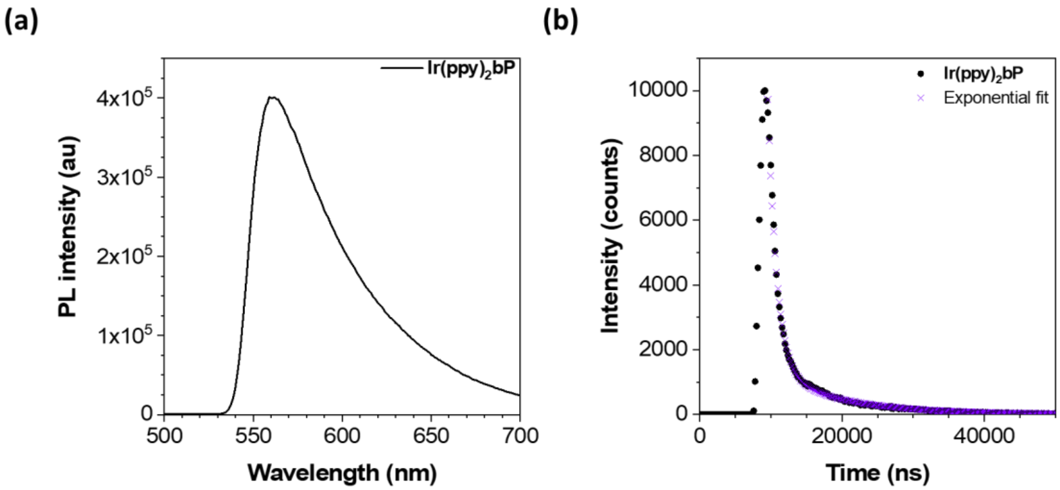

Figure S18. a) PL spectrum and b) time-resolved single photon counting (TRSPC) spectrum of **Ir(ppy)$_2$bP** in thin-film.

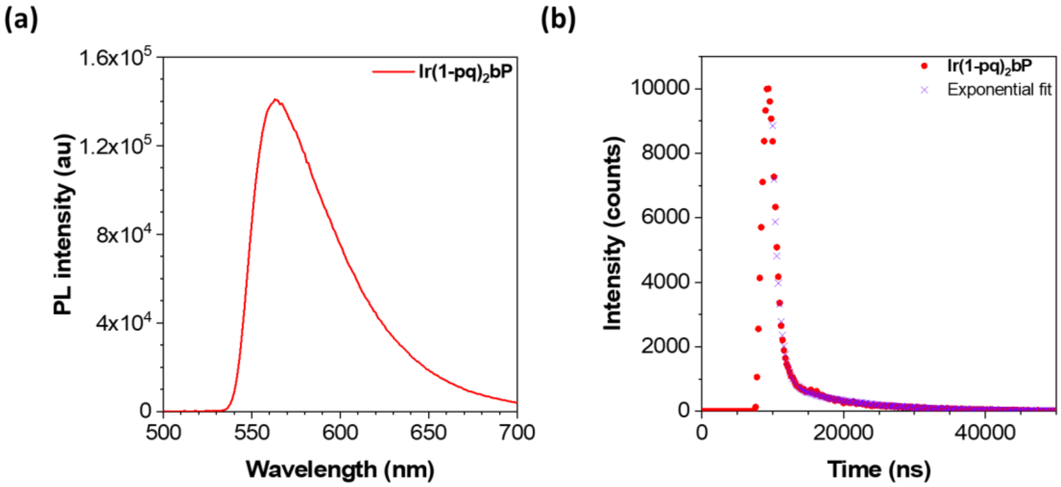

Figure S19. a) PL spectrum and b) TRSPC spectrum of **Ir(1-pq)$_2$bP** in thin-film.

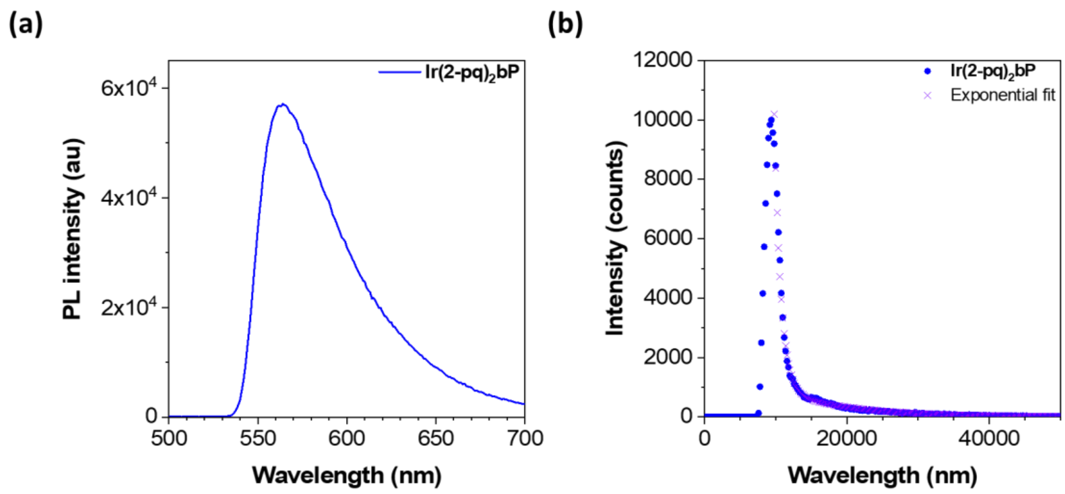

Figure S20. a) PL spectrum and b) TRSPC spectrum of **Ir(2-pq)$_2$bP** in thin-film.

References

(1) Lutkus, L. V.; Rickenbach, S. S.; McCormick, T. M. Singlet oxygen quantum yields determined by oxygen consumption. *J. Photochem. Photobiol., A* **2019**, *378*, 131–135.

(2) Norris, M. R.; Concepcion, J. J.; Glasson, C. R. K.; Fang, Z.; Lapides, A. M.; Ashford, D. L.; Templeton, J. L.; Meyer, T. J. Synthesis of Phosphonic Acid Derivatized Bipyridine Ligands and Their Ruthenium Complexes. *Inorg. Chem.* **2013**, *52*, 12492–12501.

(3) Becke, A. D. Density-functional thermochemistry. III. The role of exact exchange. *J. Chem. Phys.* **1993**, *98*, 5648–5652.

(4) Dennington, R.; Keith, T. A.; Millam, J. M. *GaussView, Version 6.1.1*; Semichem Inc., Shawnee Mission, KS, 2016.

(5) Harvey, J. N.; Aschi, M.; Schwarz, H.; Koch, W. The singlet and triplet states of phenyl cation. A hybrid approach for locating minimum energy crossing points between non-interacting potential energy surfaces. *Theor. Chem. Acc.* **1998**, *99*, 95–99.
